# Identification of three elevenin receptors and roles of elevenin disulfide bond and residues in receptor activation in *Aplysia californica*

**DOI:** 10.1038/s41598-023-34596-9

**Published:** 2023-05-11

**Authors:** Ping Fu, Yu-Shuo Mei, Wei‑Jia Liu, Ping Chen, Qing-Chun Jin, Shi‑Qi Guo, Hui‑Ying Wang, Ju‑Ping Xu, Yan-Chu-Fei Zhang, Xue‑Ying Ding, Cui-Ping Liu, Cheng-Yi Liu, Rui-Ting Mao, Guo Zhang, Jian Jing

**Affiliations:** 1 State Key Laboratory of Pharmaceutical Biotechnology, Department of Medical Psychology and Neurology, Nanjing Drum Tower Hospital, Institute for Brain Sciences, Chinese Academy of Medical Sciences Research Unit of Extracellular RNA, Jiangsu Engineering Research Center for MicroRNA Biology and Biotechnology, Advanced Institute for Life Sciences, Chemistry and Biomedicine Innovation Center, School of Life Sciences, Nanjing University, Nanjing, 210023 Jiangsu China; 2 Peng Cheng Laboratory, Shenzhen, 518000 China; 3 Department of Neuroscience and Friedman Brain Institute, Icahn School of Medicine at Mount Sinai, New York, NY 10029 USA

**Keywords:** Molecular biology, Neuroscience

## Abstract

Neuropeptides are ubiquitous intercellular signaling molecules in the CNS and play diverse roles in modulating physiological functions by acting on specific G-protein coupled receptors (GPCRs). Among them, the elevenin signaling system is now believed to be present primarily in protostomes. Although elevenin was first identified from the L11 neuron of the abdominal ganglion in mollusc *Aplysia californica*, no receptors have been described in *Aplysia*, nor in any other molluscs. Here, using two elevenin receptors in annelid *Platynereis dumerilii*, we found three putative elevenin GPCRs in *Aplysia*. We cloned the three receptors and tentatively named them apElevR1, apElevR2, and apElevR3. Using an inositol monophosphate (IP1) accumulation assay, we demonstrated that *Aplysia* elevenin with the disulfide bond activated the three putative receptors with low EC50 values (ranging from 1.2 to 25 nM), supporting that they are true receptors for elevenin. In contrast, elevenin without the disulfide bond could not activate the receptors, indicating that the disulfide bond is required for receptor activity. Using alanine substitution of individual conserved residues other than the two cysteines, we showed that these residues appear to be critical to receptor activity, and the three different receptors had different sensitivities to the single residue substitution. Finally, we examined the roles of those residues outside the disulfide bond ring by removing these residues and found that they also appeared to be important to receptor activity. Thus, our study provides an important basis for further study of the functions of elevenin and its receptors in *Aplysia* and other molluscs.

## Introduction

Neuropeptides are the most diverse class of neuromodulators that regulate a variety of physiological functions^1–7^. In order to be active, they often undergo posttranslational modification (PTM), such as forming C-terminal amidation or a disulfide bond, and exert their actions by binding to G protein-coupled receptors (GPCRs) and activating the subsequent signaling pathways^2,8,9^^.^ Together, neuropeptides and their receptors form distinct peptide signaling systems. Interestingly, it is now clear that some peptide signaling systems are present in both protostomes and deuterostomes, whereas others are only present in either protostomes or deuterostomes^1,10^. In this study, we sought to study the elevenin signaling system in *Aplysia*. Elevenin signaling system has been found to be present primarily in protostomes^1,10^. There are predicted elevenin-like receptors in urochord *Saccoglossus kowalevskii* and cephalochordate *Branchiostoma floridae*, but no elevenin precursors have been predicted in these species^2^. Thus, it is not clear if an elevenin signaling system is present in these two deuterostome species.

**Figure 1:**
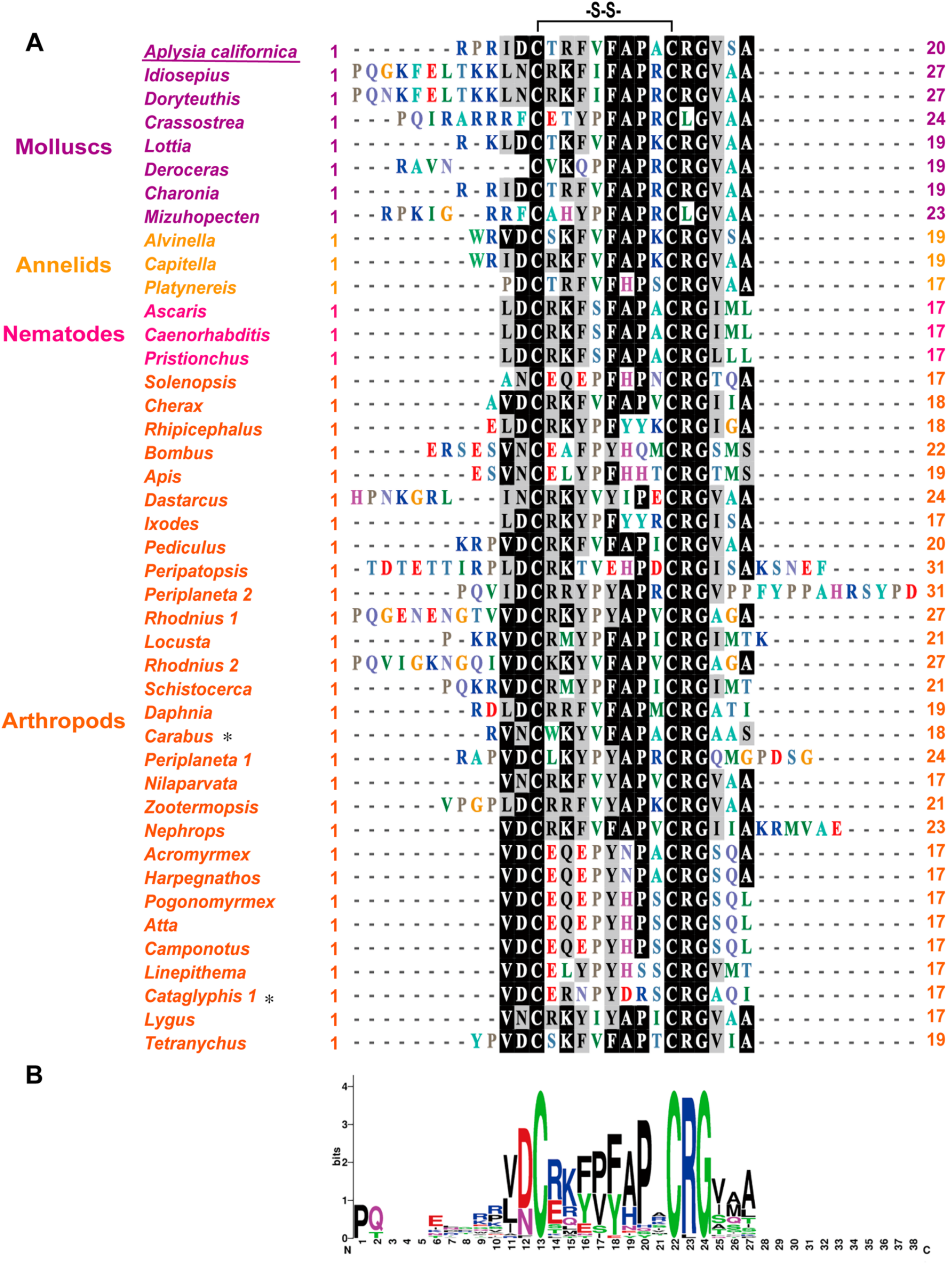
Comparison of elevenin peptides in protostomes. (**A**) Comparison of elevenin peptides from invertebrates with BioEdit (ClustalW Multiple alignment—Graphic View). * indicates that elevenins have been identified by mass spectrometry. *Aplysia californica* is underlined. (**B**) A frequency plot for the sequences listed in (**A**) using WebLogo v2.8.2 (http://weblogo.berkeley.edu/logo.cgi). See Supplementary Table 1 for the source of the sequences. –S–S– the disulfide bond. Numbers at the right denote the number of aa in a peptide.

Elevenin was first discovered in the identified neuron, L11 of the abdominal ganglion of *Aplysia*, and was named L11 neuropeptide in 1984^11^. It has 20 amino acids with two cysteine residues at positions 6 and 15, likely forming a disulfide bond. Later work in several protostome phyla, including molluscs, annelids, nematodes, and arthropods has identified or predicted the presence of similar peptides (Fig. 1). Although the number of residues in elevenins varies somewhat across species, they all share the two cysteines with 8 residues in between, and therefore these peptides were given a more generic name: elevenin^12^. Elevenin precursors have been predicted from the genomes of arthropods^9,13–18^, annelids^19^, and nematodes^20,21^. Elevenin receptors in several species have also been identified, including two elevenin receptors in annelid *Platynereis*, and arthropod *Ixodes scapularis*^2,9^.

There are a few reports on the functions of elevenins and their receptors. In one of the first studies in *Caenorhabditis elegans*, SNET-1 (an inhibitor of neprilysin-2), an elevenin homolog, is secreted as an environmental signal to inhibit the sense of smell in *C. elegans*, which can control population density^21^. In addition, an elevenin signaling system functions to control body color either through its GPCR: NlA42^22^ (although this sequence could not be located in NCBI), or the tyrosine-mediated cuticle melanism pathway^23^ in the brown planthopper, *Nilaparvata lugens*. NlA42 couples to Gq and Gs proteins, triggering PLC/Ca^2+^/PKC and AC/cAMP/PKA signaling pathways respectively in response to elevenin, which could eventually act on the tyrosine-mediated cuticle melanism pathway^23^. These are the first reports on the physiological functions of elevenin-like peptides and receptors in arthropods/insects. Moreover, in *Ixodes scapularis,* the elevenin receptor (IsElevR1) is present in type II and type III salivary glands, suggesting that elevenin and its receptor(s) may be involved in tick salivation during feeding^9^. Elevenin may also regulate digestion in *Carcinus maenas*^24^. A study in the red flour beetle *Tribolium castaneum* has found that knockdown of the cognate elevenin receptor (TC014211) using RNAi technology at the larval and pupal stages increases mortality by about 30%^25^. Finally, through immunohistochemistry, the functional analysis of ligands and receptors, and the exploration of gene expression patterns, it has been shown that the elevenin signaling pathway plays a subtle role in promoting ovarian development in *Tribolium*^9^.

Despite the progress described above, few studies have been carried out on molluscs, particularly on the receptors. In the sea slug *Plakobranchus ocellatus*, researchers have cloned a segment of a putative elevenin receptor^26^, but no complete elevenin receptor has been found in any molluscs. We aimed to explore the elevenin receptors in *Aplysia*. *Aplysia* has been an experimentally advantageous model system for studies of motivated behaviors^27–40^, learning and memory^41–45^, and neuromodulation^6,46^, including neuropeptides^47–58^ and receptors^2,3,8,59–62^. Here, we used bioinformatics, molecular and cellular approaches to identify three putative elevenin receptors. We then showed that elevenin can activate all three receptors using a cellular assay. We also showed the importance of the disulfide bond and some of the conserved residues of elevenin in the activation of the receptors. This is the first identification of elevenin receptors in molluscs and provides an important basis for further study of the physiological functions of elevenin and its receptors in *Aplysia*.

## Results

### Molecular characterization and phylogenetic analysis of Elevenin

**Figure 2:**
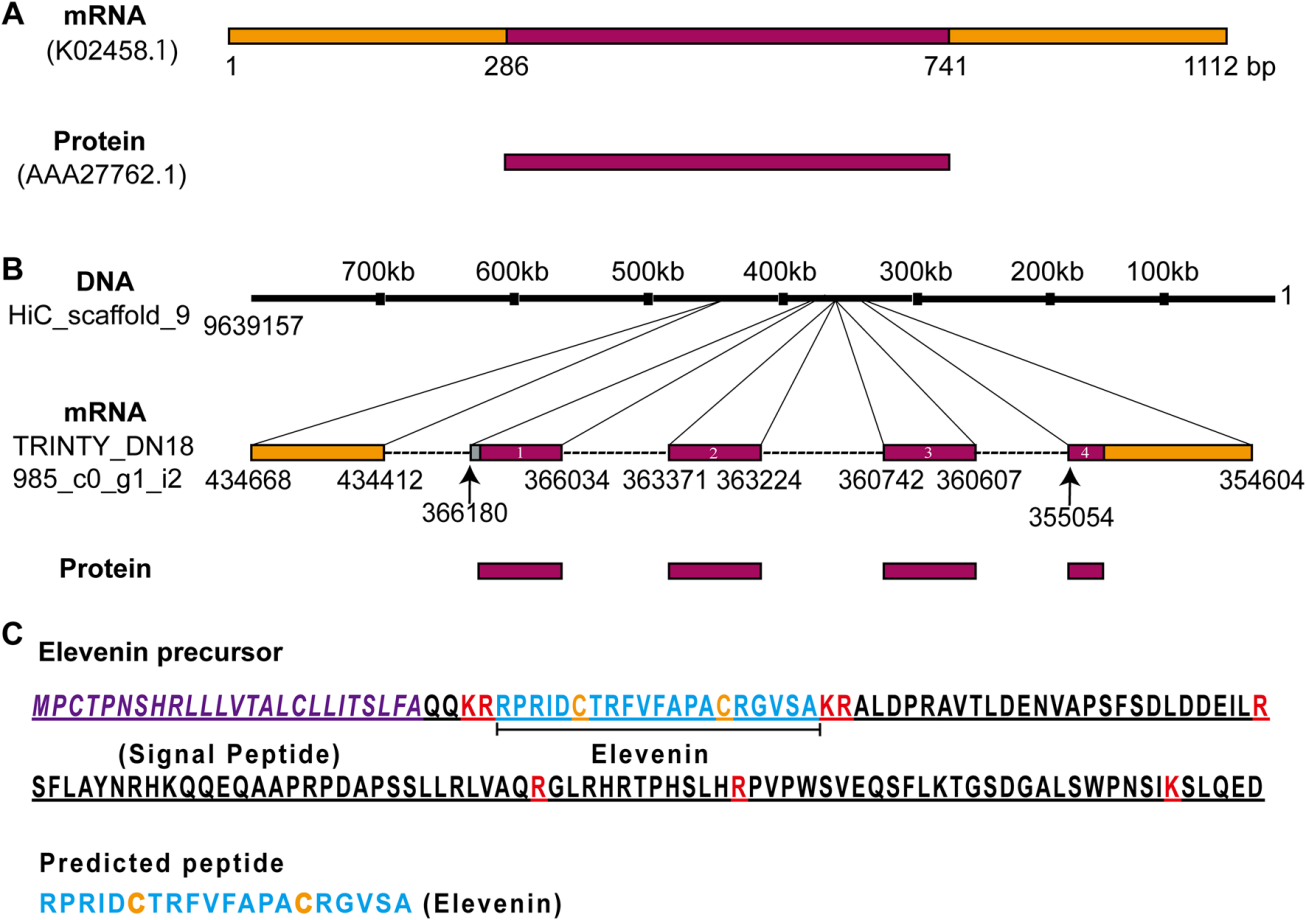
Gene expression mapping of the *Aplysia* elevenin precursor. (**A**) An mRNA from NCBI (K02458), named *Aplysia* neuron L11 neuropeptide mRNA^11^. (**B**) DNA HiC_scaffold_9 (Note that the nucleotide number on top starts from the right) from *Aplysia* gene nucleotide databases (the AplysiaTools) and the protein generated from this mRNA is the same as those in (**A**), Numbering in the mRNA (**B**) denotes the exon number. (**C**) The complete protein sequence of the *Aplysia* elevenin precursor contains a signal peptide (shown in purple) and a mature elevenin peptide (shown in blue). Potential basic cleavage sites are shown in red. The two cysteines that likely form a disulfide bond are shown in orange.

In a previous study^11^, an mRNA with a length of 1112 bp (NCBI accession number: k02458.1) has been cloned from L11 neurons in *Aplysia*, and it contains a complete CDS sequence for a precursor for a 151 aa neuropeptide (accession number: AAA27762.1) (Fig. 2A). A mRNA sequence (TRINITY_DN18985_c0_g1_i2) was also found in AplysiaTools database (Fig. 2B), which differs from k02458.1 in one nucleotide, but the protein it encodes is identical to AAA27762.1.

**Figure 3:**
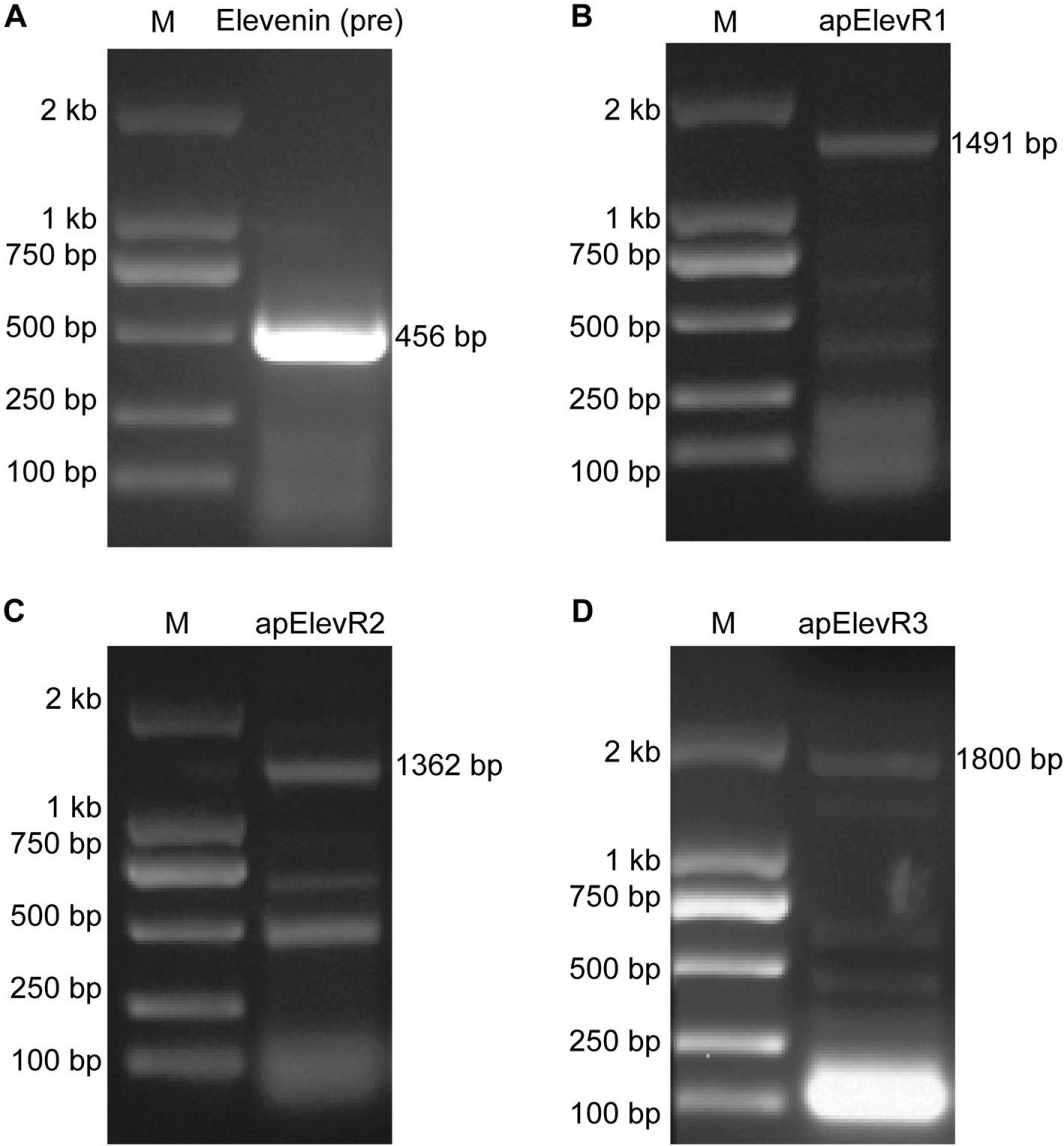
Cloning of the *Aplysia* elevenin precursor and putative receptors. (**A**) A PCR product of the elevenin precursor (pre) with a length of 456 bp from the *Aplysia* abdominal ganglion. (**B**) A PCR product for a putative receptor (apElevR1) with a length of 1491 bp. (**C**) A putative receptor (apElevR2) with a length of 1362 bp was cloned by PCR. (**D**) A PCR product of a complete putative receptor (apElevR3) with a length of 1800 bp. *M* marker.

We used NeuroPred^63^ to predict the possible elevenin peptide from the *Aplysia* elevenin precursor (Fig. 2C). One putative mature peptide with 20 amino acids was produced: RPRIDCTRFVFAPACRGVSA, which contains two cysteines separated by 8 amino acids. We compared the *Aplysia* elevenin peptide with elevenins in other molluscs, annelids, nematodes, and arthropods (Fig. 1, see Supplementary Table 1 for information on these sequences). Cys, Gly, Arg, Pro, and Asp residues appear to be highly conserved (Fig. 1A), whereas the total number of residues in elevenins between different species varied from 19 to 27. In addition, we generated a frequency plot (Fig. 1B) of single residues in the peptides (http://weblogo.berkeley.edu/logo.cgi). Both the alignment results (Fig. 1A) and the frequency plot (Fig. 1B) showed the same conserved residues. We verified the expression of the mRNA encoding elevenin precursor by performing RT-PCR on the abdominal ganglion from *Aplysia* (Fig. 3A, see Supplementary Fig. 1A for the complete gel, primer sequences are in Supplementary Table 2). As expected, a 456 bp fragment (identical to TRINITY_DN18985_c0_g1_i2 in the AplysiaTools database) encoding the 151 aa precursor could be amplified. We also tried other ganglions, including cerebral, buccal, and pedal ganglions, but could not obtain this sequence, supporting that elevenin mRNA may be primarily expressed in the abdominal ganglion.

**Figure 4:**
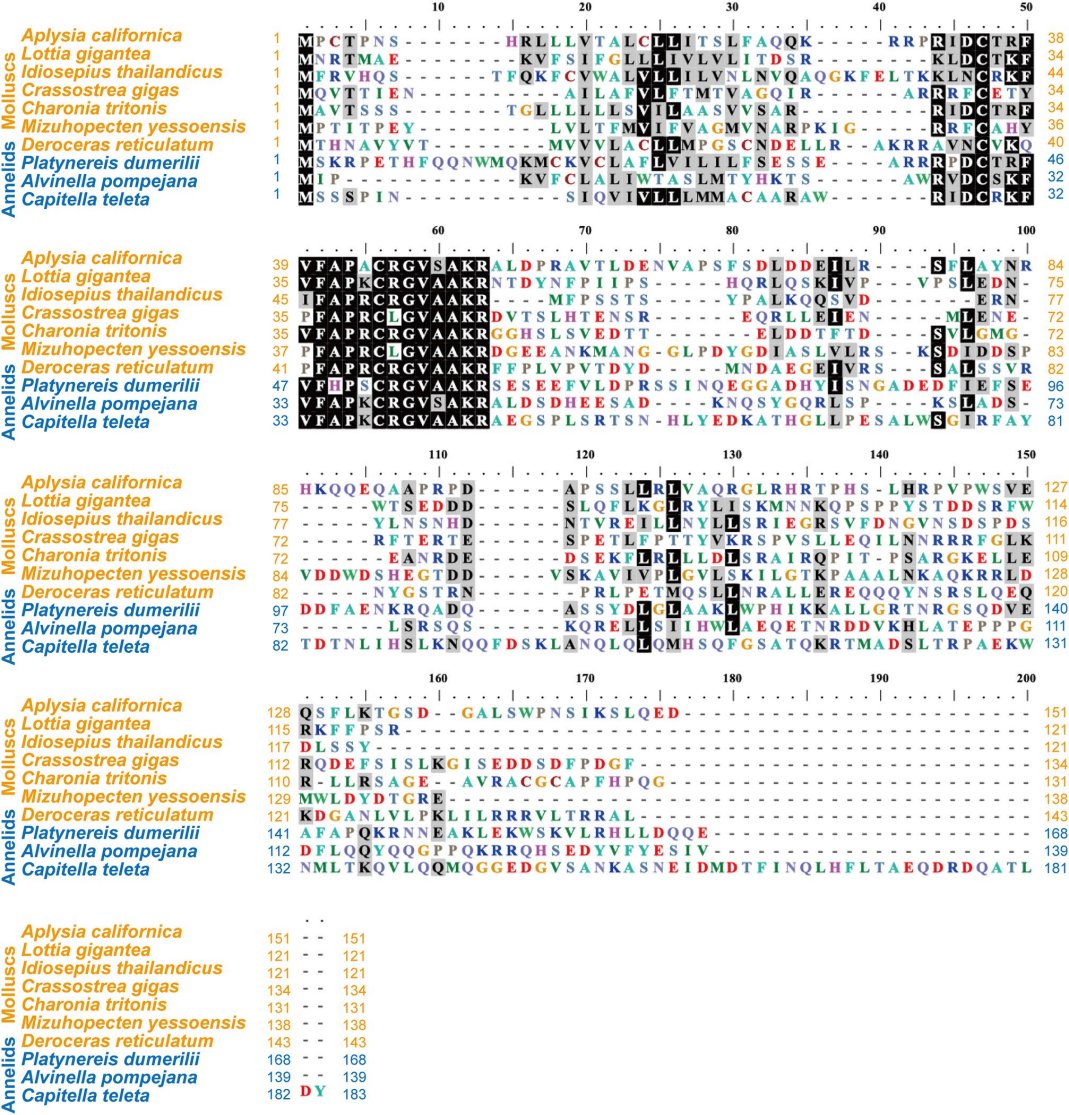
The *Aplysia* elevenin precursor vs. similar precursors in lophotrochozoans. Comparison of the *Aplysia* elevenin precursor vs. similar precursors in lophotrochozoa (i.e., molluscs and annelids) using BioEdit v5.0.6 (ClustalW Multiple alignment—Graph view).

Next, we compared identities and similarities between the *Aplysia* elevenin precursor and other elevenin precursors in selected invertebrate species (Table 1, see Supplementary Table 3 for information on these sequences). The *Aplysia* elevenin precursor is most closely related to that of *Platynereis* with a similarity of 41.52%. Furthermore, we compared 7 elevenin precursors in molluscs with 3 in annelids with BioEdit (Fig. 4), which showed that the regions for the elevenin peptide sequences are the only portions that are highly conserved and indicated that no other peptide is encoded by these precursors.

**Table 1:** Identities and similarities of the elevenin precursor from *Aplysia* with elevenin precursors from other molluscs, annelids, nematodes, and arthropods using BioEdit (pairwise alignment-calculate identity/similarity for two sequences).

Similarity matrix: BLOSUM62	Elevenin precursor (*Aplysia californica*)	
Name	Identities (%)	Similarities (%)
Molluscs		
Elevenin [*Deroceras reticulatum*]	20.78	37.01
Elevenin [*Lottia gigantea*]	20.53	37.09
Elevenin [*Crassostrea gigas*]	19.87	37.75
Elevenin [*Idiosepius thailandicus*]	18.47	31.21
Elevenin [*Mizuhopecten yessoensis*]	17.88	33.77
Elevenin [*Charonia tritonis*]	12.58	25.17
Annelids		
Elevenin [*Platynereis dumerilii*]	24.56	41.52
Elevenin [*Alvinella pompejana*]	24.34	34.87
Elevenin [*Capitella teleta*]	20.63	34.92
Nematodes		
Elevenin [*Pristionchus pacificus*]	16.56	30.46
Elevenin [*Caenorhabditis elegans*]	13.91	25.17
Arthropods		
Elevenin [*Nilaparvata lugens*]	20.53	30.46
Elevenin [*Carabus violaceus*]	19.11	35.03
Elevenin [*Schistocerca gregaria*]	18.54	31.79
Elevenin [*Nephrops norvegicus*]	17.22	32.45
Elevenin [*Bombus terrestris*]	17.11	34.87
Elevenin [*Rhipicephalus microplus*]	16.56	32.45
Elevenin [*Apis mellifera*]	15.13	34.21
Elevenin [*Lygus Hesperus*]	14.47	25.00
Elevenin [*Cataglyphis nodus*]	13.25	27.15
Elevenin [*Cherax quadricarinatus*]	17.88	33.77
Elevenin [*Ixodes scapularis*]	17.88	30.46
Elevenin [*Locusta migratoria*]	17.88	31.12

### Identification of three putative elevenin receptors in *Aplysia*

**Figure 5:**
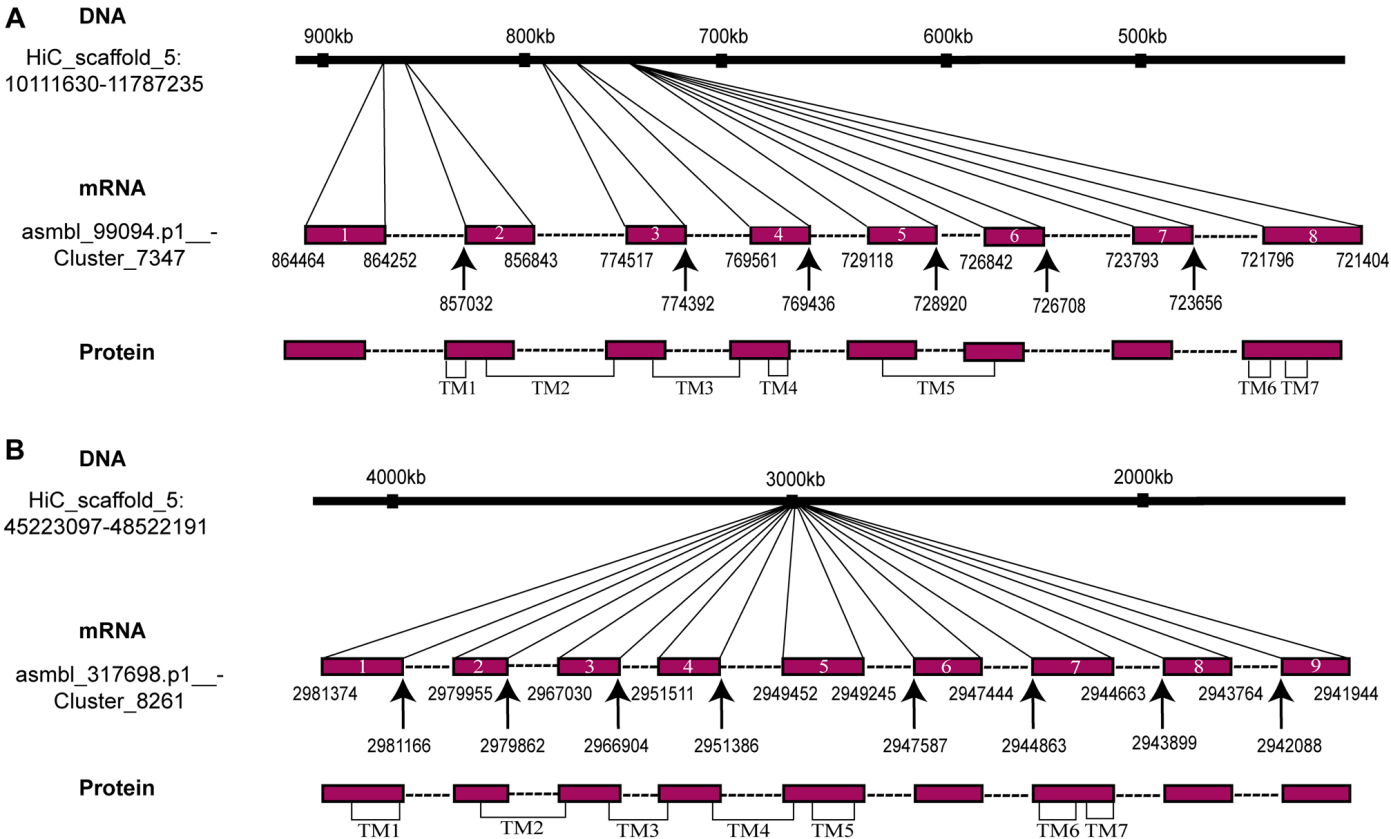
Gene expression mapping of the *Aplysia* apElevR1 and apElevR2. (**A**) DNA HiC_scaffold_5: 10,111,630–11,787,235 (apElevR1) from *Aplysia* gene nucleotide databases (the AplysiaTools). (**B**) DNA HiC_scaffold_5: 45,223,097–48,522,191 (apElevR2) from *Aplysia* gene nucleotide databases (the AplysiaTools). Numbering in the mRNA denotes the exon number. The approximate locations of the transmembrane domain (TM) 1–7 are indicated for the proteins.

**Figure 6:**
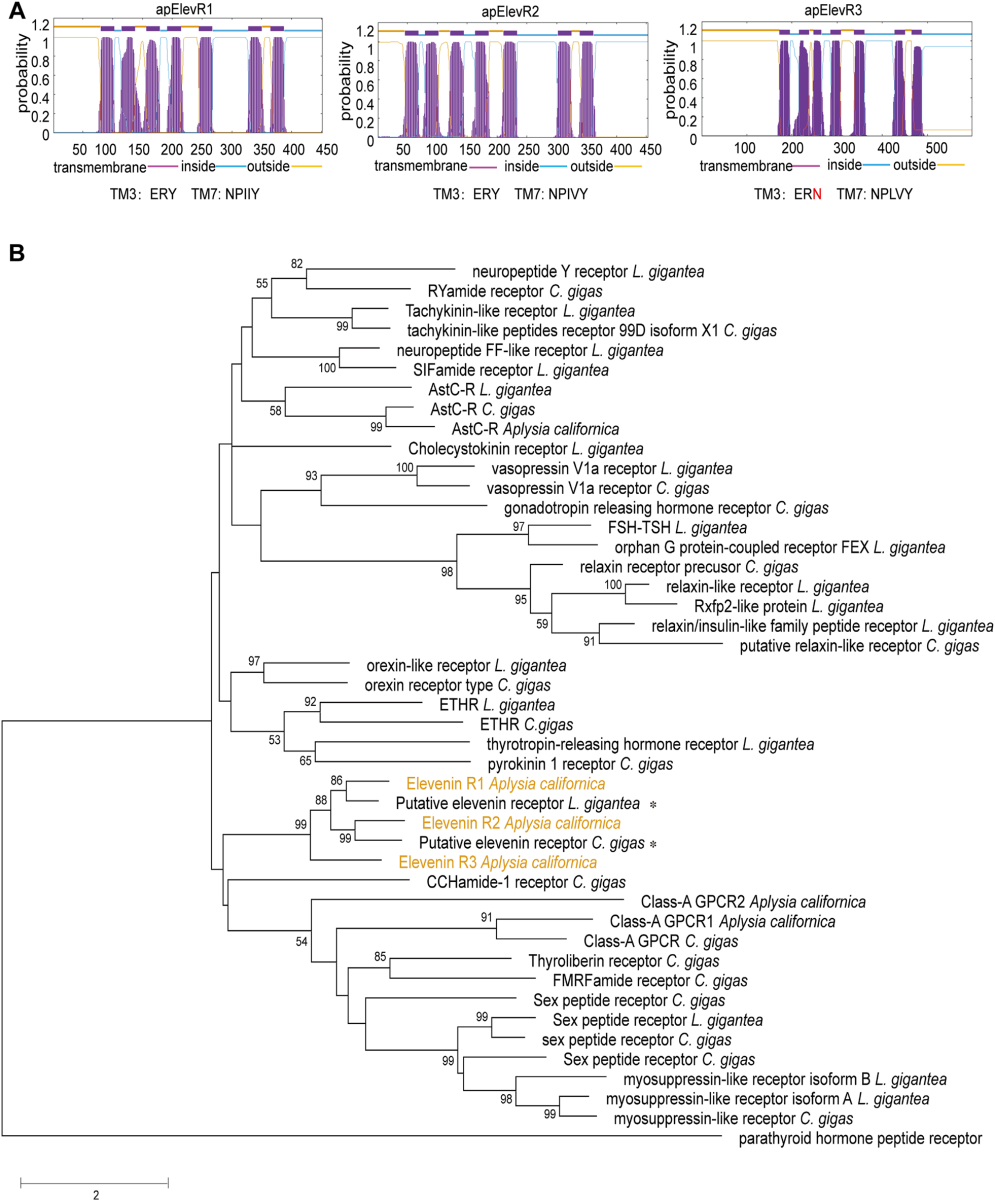
Bioinformatics of putative elevenin receptors. (**A**) Prediction of seven transmembrane domains of putative receptors: apElevR1, apElevR2, apElevR3 using TMHMM 2.0 (https://dtu.biolib.com/DeepTMHMM). Conserved motifs in transmembrane domain 3 (TM3, D/ERY) and TM7 (NPXXY) are shown. The last residue, N, of TM3 motif for apElevR3 is shown in red because it is not conserved. (**B**) A phylogenetic tree of the three *Aplysia* proteins, apElevR1, apElevR2, apElevR3 (shown in orange), and two putative elevenin receptors (marked by *) of *L. gigantea* and *C. gigas* with a number of Class A GPCR sequences derived from our previous work^8^. The tree is drawn to scale, with branch lengths measured in the number of substitutions per site. Numbers at the nodes are bootstrap values as a percentage. Only bootstrap values greater than 50 are shown.

Currently, there are no complete elevenin receptors that have been identified in any mollusc. To identify putative elevenin receptors in *Aplysia*, we used the sequences of the two deorphanized elevenin receptors from the annelid *Platyneris dumerilii* (the first identified elevenin receptors) to perform a BLAST search of NCBI GenBank. A total of 4 *Aplysia* sequences (XM_005096018.3, XM_035970075.1, XM_013090833.1, XM_035970908.1) were found. The sequence (XM_013090833.1) is an incomplete sequence for the *Aplysia* leucokinin-like receptor (ALKR) that we have identified recently (NCBI: OP292655.1)^61^. The three *Aplysia* NCBI mRNA sequences were incomplete, but we found complete sequences for all three in the AplysiaTools database (Supplemental Table 4). The predicted intron–exon structures of two of the receptors were shown in Fig. 5, whereas no genomic information is available for the third receptor in the AplysiaTools database because the database is still evolving and might not be complete at this point. The 7TM and conserved motifs of the three receptors were analyzed using the NCBI Conserved Domain Database (https://www.ncbi.nlm.nih.gov/Structure/cdd/wrpsb.cgi) and TMHMM 2.0 (https://dtu.biolib.com/DeepTMHMM) (Fig. 6A). The similarities between apElevR1, apElevR2, apElevR3, and the two receptors in *Platynereis* are approximately 54%, 55%, and 40% respectively (see the Clustal table in Supplementary Table 4), therefore we putatively named them elevenin receptors (apElevR1-3). We cloned apElevR1, apElevR2, and apElevR3 from the *Aplysia* CNS cDNA (Fig. 3B-D, see Supplementary Fig. 1B,C for complete gels, primer sequences are provided in Supplementary Table 2). The PCR products were found to produce identical proteins to those in the AplysiaTools. We do note that although apElevR1 and apElevR3 mRNA sequences are identical to those in AplysiaTools, apElevR2 does differ from the AplysiaTools sequence in one nucleotide, but this single nucleotide polymorphism (SNP) did not affect the protein sequence. The gene sequences have been deposited into the NCBI database (apElevR1 GenBank accession number: OQ686759; apElevR2 GenBank accession number: OQ686760; apElevR3 GenBank accession number: OQ686761). We also compared *Aplysia* putative elevenin receptors with elevenin receptors from several species including those from *Platynereis* and *Ixodes* using BioEdit (Supplementary Fig. 2).

**Figure 7:**
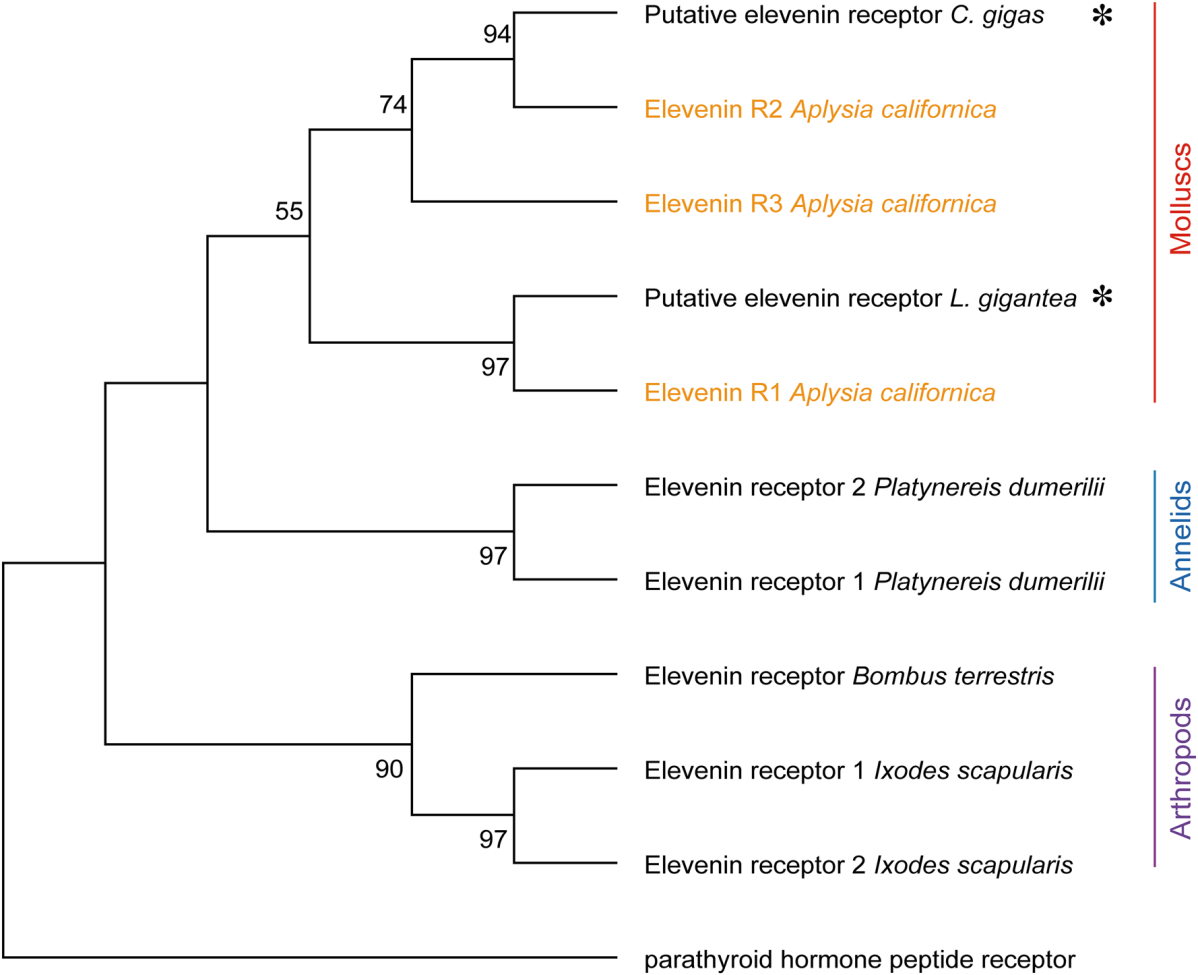
A phylogenetic tree of elevenin receptors in invertebrates*.* The tree with predicted or verified elevenin receptors in invertebrates was generated using MEGA X with 1000 replicates. This phylogenetic tree indicates that *Aplysia* elevenin receptors are more closely related to sequences in mollusc *L. gigantea* and *C. gigas*. * indicates that the receptors are predicted. Numbers at the nodes are bootstrap values as percentages. Only bootstrap values greater than 50 are shown.

To understand the phylogenetic relationship of the three putative elevenin receptors, we first took advantage of a number of different Class-A GPCR sequences primarily from mollusc *Crassostrea gigas* and *Lottia gigantea* for a phylogenetic tree generated in our previous work (For the sequence information, see their Supplementary Table 4)^8^. Then we added the three putative *Aplysia* elevenin receptor sequences, together with predicted elevenin receptors in *L. giagantea* and *C. gigas* (see Supplementary Table 4). The updated phylogenetic tree (Fig. 6B) shows that the three putative elevenin receptors of *Aplysia* are clustered with elevenin receptors of* L. giagantea* and *C. gigas*, supporting that they are likely elevenin receptors. Finally, we generated a phylogenetic tree with only the elevenin receptors from *Aplysia*, and other molluscs, annelids, and arthropods (Fig. 7). The three putative elevenin receptors in *Aplysia* are clustered with molluscan receptors.

### Activation of putative receptors by the elevenin peptide and roles of specific residues in elevenin

**Figure 8:**
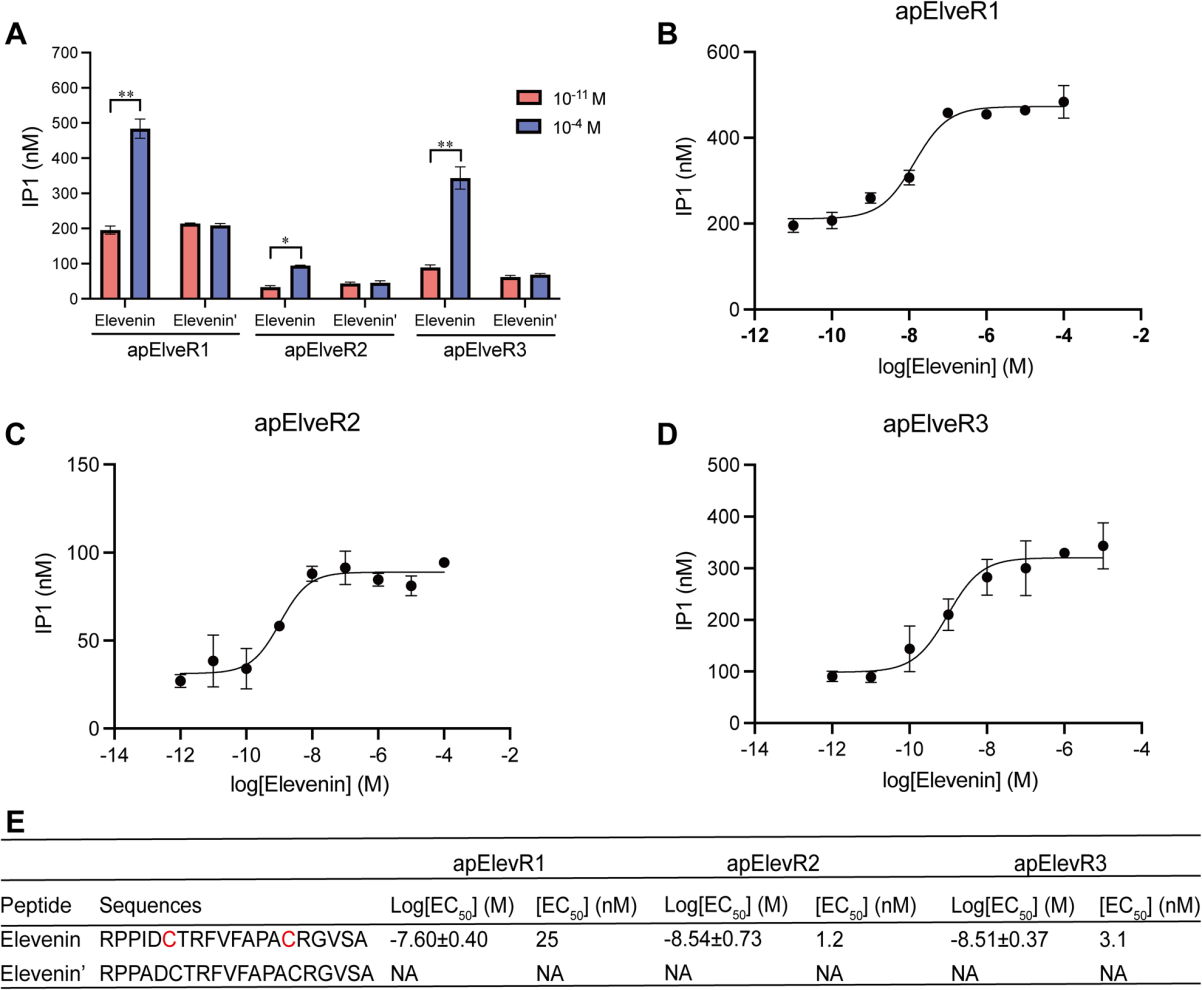
Activation of the three putative receptors by *Aplysia* elevenin as demonstrated using an IP1 accumulation assay. (**A**) Screening of elevenin putative receptors using IP1 assay with two different concentrations (10^–11^ M and 10^–4^ M). (**B-D**) Dose–response curves of the activation of apElevR1, apElevR2, and apElevR3 by elevenin. (**E**) Sequences with (Elevenin) or without the disulfide bond (Elevenin') and a summary of the average log[EC_50_] and EC_50_ values on apElevRs. Cysteines with the disulfide bond are shown in red. n = 3 for all the tests. Student’s t-test, *P < 0.05, **P < 0.01. Error bar: mean ± SEM.

To determine whether the *Aplysia* elevenin can activate the putative elevenin receptors, we cloned apElevRs to pcDNA3.1 (+) plasmid and transfected the three apElevRs in CHO-K1 cells. We used an IP1 accumulation assay to measure the concentration of IP1, which is a by-product in the Gαq signaling pathway, to provide evidence for the activation of receptors by elevenin. Similar to previous work in *Platynereis*^2^, we did not need to co-transfect a promiscuous Gαq protein (also known as Gα16) to obtain an IP1 response. This indicates that the three *Aplysia* elevenin receptors could couple to the Gαq protein endogenously present in CHO cells. Initially, we used the elevenin peptide at two concentrations of 10^–11^ M and 10^–4^ M to screen apElevR1, apElevR2, and apElevR3. The results showed that 10^–4^ M elevenin showed significantly higher activation on all three receptors compared with 10^–11^ M, supporting that these three receptors are true receptors for elevenin (Fig. 8A). We also used synthetic elevenin peptide without the disulfide bond and found that elevenin without the disulfide bond could not activate the receptors, suggesting the importance of the disulfide bond for the activity of receptor (Fig. 8A, and E). Further, we used different concentration gradients of elevenin, ranging from 10^–12^ to 10^–4^ M, to generate the dose–response curves (Fig. 8B–E). The EC_50_ values of the three elevenin receptors were 25, 1.2, and 3.1 nM respectively.

**Figure 9:**
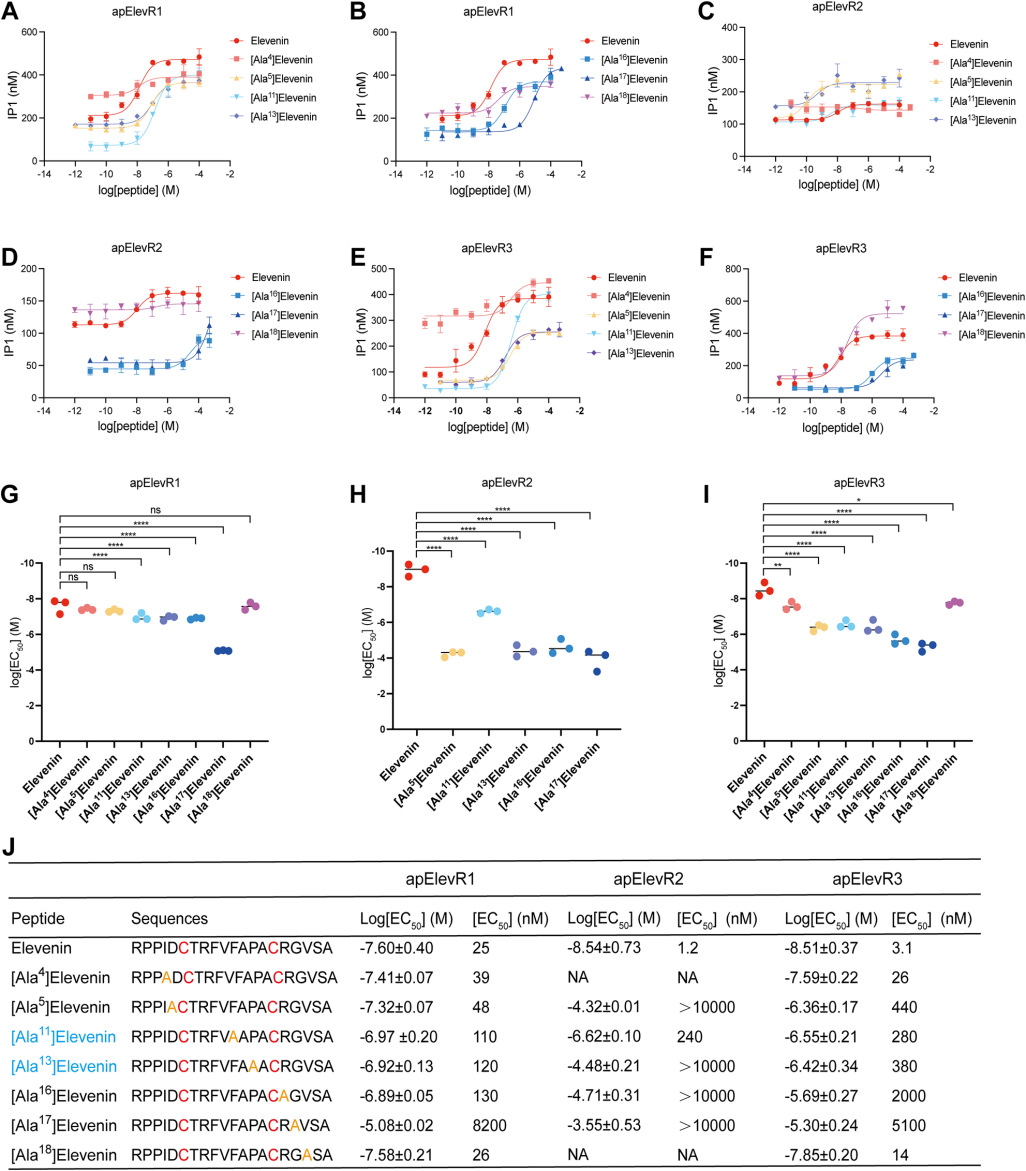
Effects of the *Aplysia* elevenin analogs with alanine substitution on the three putative receptors. (**A-F**) Representative examples of dose–response curves of the activation of apElevR1, apElevR2, and apElevR3 with substitution of a conserved amino acid of elevenin with ala. (**G-I**) The log[EC_50_] values of apElevR1, apElevR2, and apElevR3 after replacing different conserved amino acids of elevenin with ala (most conserved amino acids included I4, D5, F11, P13, R16, G17, and V18 as predicted by Weblogo v2.8.2), One-way ANOVA, F (7, 16) = 80.34, *P* < 0.0001 (**G**), F (5, 12) = 88.51, *P* < 0.0001 (**H**), F (7, 16) = 55.08, *P* < 0.0001 (**I**). Bonferroni post-hoc test: *P < 0.05, **P < 0.01, ****P < 0.0001. Error bar: mean ± SEM. (**J**) Sequences of all peptides tested and a summary of the average log[EC_50_] and EC_50_ values on apElevRs. n = 3 for all the tests. Cysteines in red represent the disulfide bond in the peptide. Alanine in orange denotes the substitution of each residue in the peptide analogs. Analogs with substituted residues inside the disulfide bond were highlighted in blue.

We have shown that C6, C15, G17, R16, P13, D5, F11, V18, and I4 in elevenins appear to be highly conserved (Fig. 1). To determine the potential importance of these conserved residues in receptor activity, we synthesized analogs by replacing these amino acids, G17, R16, P13, D5, F11, V18, and I4 (except Cys) with Ala, respectively. The results of the IP1 accumulation assay showed that the log[EC_50_] and EC_50_ values of the elevenin receptors by most of the analogs increased significantly, and the degree of effects was approximately G17 > R16 > P13 > D5 > F11 > V18 > I4, particularly for apElevR1 (Fig. 9). Specifically, apElevR1 and apElevR3 had similar results for the peptide analogs with different conserved amino acids replaced with Ala. For apElevR1, the log[EC_50_] values of [Ala^11^]Elevenin, [Ala^13^]Elevenin, [Ala^16^]Elevenin, and [Ala^17^]Elevenin were significantly increased compared with elevenin (Fig. 9A,B,G and J). Moreover, the log[EC_50_] of [Ala^4^]Elevenin, [Ala^5^]Elevenin, and [Ala^18^]Elevenin had no significant difference when compared with elevenin (Fig. 9A,B,G and J). ApElevR3 can be activated by the seven elevenin analogs at various degrees, and the log[EC_50_] values of the seven analogs increased significantly compared with elevenin (Fig. 9E,F,I and J). In contrast, all six analogs (except [Ala^11^]Elevenin) have dramatically higher log[EC_50_] values for apElevR2, suggesting apElevR2 is similarly sensitive to the substitution of either of the six residues (Fig. 9C,D,H and J). These results demonstrated that the conservation of amino acids plays an important role in elevenin activity (Table 2).

**Table 2:** The EC_50_ values on apElevRs by elevenin analogs with alanine substitutions. Analogs are ordered based on the conserveness of each residue (Fig. 1).

Conserved residues	apElevR1	apElevR3	apElevR2
Elevenin	25	3.1	1.2
[Ala^17^]Elevenin	8200	5100	> 10,000
[Ala^16^]Elevenin	130	2000	> 10,000
*[Ala*^*13*^*]Elevenin*	*120*	*380*	> *10,000*
[Ala^5^]Elevenin	48	440	> 10,000
*[Ala*^*11*^*]Elevenin*	*110*	*280*	*240*
[Ala^18^]Elevenin	26	14	NA
[Ala^4^]Elevenin	39	26	NA

Analogs with substituted residues inside the disulfide bond were highlighted in italic.

**Figure 10:**
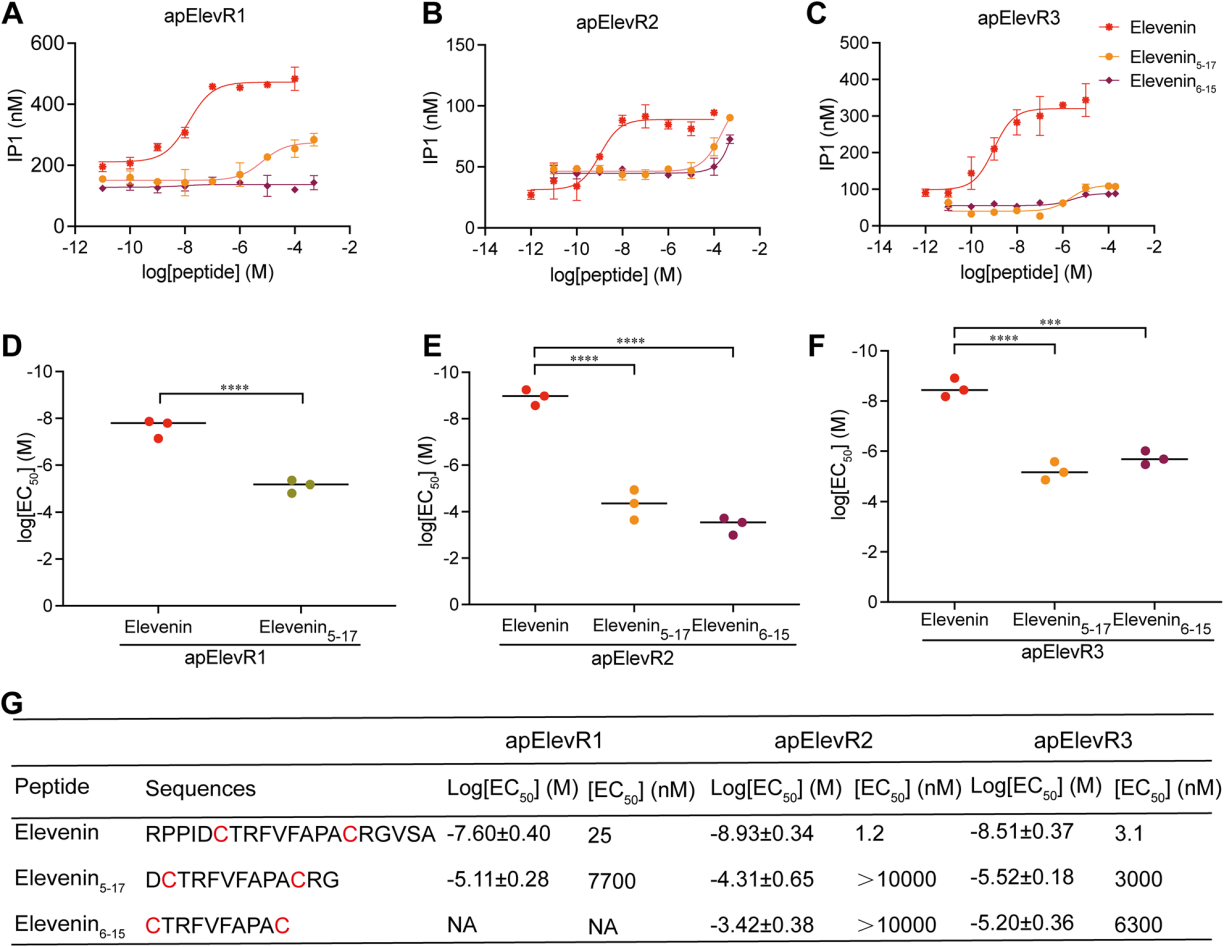
Effects of residue deletions of elevenin on receptor activation. (**A-C**) Representative examples of dose–response curves of the activation of apElevR1, apElevR2, and apElevR3 with different lengths of elevenin. (**D-F**) The log[EC_50_] values of apElevR1, apElevR2, and apElevR3 with different lengths of elevenin. Unpaired T-test, ****P < 0.0001 (**D**). One-way ANOVA, F (2, 6) = 115.6, *P* < 0.0001 (**E**), F (2, 6) = 82.91, *P* < 0.0001 (**F**). Bonferroni post-hoc test for **E** and **F**: ***P < 0.001, ****P < 0.0001. Error bar: mean ± SEM. (**G**) Sequences of all peptides tested and summary of the average log[EC_50_] and EC_50_ values on elevenin receptors. n = 3 for all the tests.

In Fig. 8A, we showed that three receptors can't be activated by elevenin without a disulfide bond. We sought to investigate whether sequences other than the disulfide bond might play some roles in receptor activity. Thus, we synthesized two truncated elevenin analogs with different lengths and kept sequences between cysteines the same: Elevenin_5-17_ and Elevenin_6-15_. Elevenin_5-17_ has the conserved D5, R16, and G17 outside of two cysteines, whereas elevenin_6-15_ only has the sequence between the cysteines. The three receptors responded differently to the two truncated elevenin peptides. The log[EC_50_] values of the truncated elevenin_5-17_ and elevenin_6-15_ were all significantly increased when compared with that of elevenin for the three receptors. For apElevR1 and apElevR3, the log[EC_50_] values of elevenin_6-15_ were increased significantly compared with those for elevenin_5-17_ (Fig. 10A,C,D,F, and G). In fact, elevenin_6-15_ apparently did not have any effect on apElevR1. For apElevR2, both analogs had higher log[EC_50_] values compared with that for elevenin (Fig. 10B,E, and G). This is consistent with the effects of residue substitution experiments on apElevR2 (Fig. 9C,D,H and J), indicating that apElevR2 is highly sensitive to any alternations in elevenin. Thus, the amino acids outside of the disulfide bond also appear to be important for the elevenin peptide to retain its activity on the receptors.

### Putative expression levels of the elevenin precursor and receptors

Finally, we characterized the expression of elevenin and its receptors using an NCBI database from a broad spectrum of RNA-seq data (GSE79231) obtained from *Aplysia* adult and developmental stages^64^. The highest level of the elevenin precursor (NM_001204550.1) expression was found in the CNS (Supplementary Fig. 3A). ApElevR1 (XM_005096018.2) was primarily expressed in statocyst as well as mantle and gills (Supplementary Fig. 3B) with relatively weak expression in the CNS. ApElevR2 (XM_013083173.1) was also expressed most highly in statocyst, and with the second high-level expression in the CNS (Supplementary Fig. 3C). Note that XM_013083173.1 (apElevR2) is incomplete and has been replaced by the accession number of XM_035970075.1 in NCBI, which is still incomplete (see the section “Identification of three putative elevenin receptors in *Aplysia*”). In addition, no information is available on apElevR3 (XM_035970908.1).

## Discussion

In previous work, elevenin was initially identified as a cDNA sequence encoding L11 neuropeptide precursor from *Aplysia*^11^*.* Here, we verified the elevenin precursor by nucleic acid sequencing, and more importantly, we identified three elevenin receptors in *Aplysia* for the first time and demonstrated the roles of the disulfide bond and conserved amino acid residues of the elevenin ligand on receptor activity through bioinformatics and cell-based assay.

### Elevenin peptides and their precursors

After the elevenin precursor was first discovered in L11 neurons of *Aplysia*^11^, homologous precursors have subsequently been found in other molluscs and *C.elegans*, and therefore they were renamed elevenin^12^. Later, an elevenin precursor was identified by sequencing the cDNA in *Ixodes*^9^. Subsequent bioinformatic work has predicted the presence of the elevenin precursor and peptides in more species, including molluscs, annelids, nematodes, and arthropods (Fig. 1). Consistent with the previous work, we were able to clone the *Aplysia* elevenin precursor from the abdominal cDNA (Fig. 3A). It encodes a 20 amino acid peptide if cleavage is done after the first Arg residue following the signal peptide.

A few studies have identified the mature forms of elevenins in two species (*Carabus violaceus* and *Cataglyphis nodus*). Specifically, the elevenin peptide likely with the disulfide bond was identified by mass spectrometry (MS) in the brains of *Cataglyphis nodus*^18^. In addition, it was noted that the elevenin in *Carabus violaceus* has been identified by MS, but a clear MS spectrum was not obtained, although there were ion signals in mass fingerprints^65^. Interestingly, conopeptides in the venoms of cone snails contain peptides often with two or more cysteines^66^. Indeed, venoms of cone snails include precursors that encode peptides similar to elevenins, and the mature elevenin-like peptide with a disulfide bond in the Australian cone snail *Conus victoriae*, named elevenin-Vc1, was identified by MS^67^. Thus, although it remains to be determined experimentally in more species, we expect that the mature forms of endogenous elevenins in protostomes are likely to form a disulfide bond. This is supported by our finding that elevenin without the disulfide bond cannot activate the elevenin receptors at all in *Aplysia* (Fig. 8).

### Identification of three *Aplysia* elevenin receptors

Although the function of elevenin was first studied in *C.elegans*, its actual elevenin receptors have not yet been found in this species. In fact, prior to our work, there are a total of 6 elevenin receptors that have been described (Fig. 7). First, there are two GPCRs that have been deorphanized as elevenin receptors in both annelid *Platynereis* and arthropod *Ixodes*^2,9^^.^ There is a single elevenin receptor in *Nilaparvata lugens* or *Bombus terrestris*^23^ although we could not find the sequence for *Nilaparvata* NlA42 in NCBI. Second, in annelid *Platynereis*, the EC_50_ values of two elevenin receptors are 62 nM and 1.3 nM. The two receptors are activated by elevenin with EC_50_ values of 0.01 nM and 5.59 nM in *Ixodes*.

In molluscs, before this present work, there is only one study that identified a partial sequence of a putative elevenin receptor in sea slug *Plakobranchus ocellatus*^26^, which appeared to contain only 2 transmembrane domains based on our analysis. In our study, we identified three complete elevenin receptors in *Aplysia*. The EC_50_ values of the three receptors apElevR1, apElevR2, and apElevR3 in response to elevenin with the disulfide bond were 25 nM, 1.2 nM, and 3.1 nM, respectively (Fig. 8), which are in the similar ranges to the EC_50_ values for receptors in the *Platynereis* and *Ixodes*. Moreover, for the assessment of receptor activity of all elevenin receptors in the three species, an expression system, i.e., CHO cells, is used and a promiscuous Gαq protein (also known as Gα16) was not needed to obtain a response for IP1 (*Aplysia*) or Ca^2+^ (*Platynereis* and *Ixodes*), both of which are generated in the Gq signaling pathway. Thus, all elevenin receptors identified so far could couple to the Gαq protein endogenously present in CHO cells. However, it remains to be determined whether these receptors could also couple to other Gα proteins, such as Gαs or Gαi.

Overall, our work is the first time to explicitly identify the complete elevenin receptors in a mollusc. Given that currently, only *Aplysia* has three elevenin receptors, whereas other species had at most two^2,9^, an open question is whether the three elevenin receptors are only present in molluscs, or whether there may be additional receptors that remained to be discovered in other species, e.g., in annelid *Platynereis*. This issue could be resolved as more elevenin receptors are being characterized in protostomes in the future. It is also interesting to note that phylogenetic trees (Figs. 6 and 7) suggest that each of apElevR1, apElevR2, and apElevR3 might constitute a separate clade with putative orthologs of other molluscan species (*C. gigas* and *L. gigantea*), which differ from the two receptors in *I. scapularis*. The results imply that at least three elevenin receptors might have been generated in a common ancestor of molluscs, and are conserved in each current mollusc, although some of them might have been deleted or become pseudogenes during their evolutionary processes. In contrast, the two *Ixodes* elevenin receptors could be generated by duplication in the evolutionary lineage of *Ixodes*. As elevenin receptors are being identified in more species, we will have a better idea of whether these evolutionary insights hold.

### Role of elevenin disulfide bond and conserved residues in receptor activation

We showed that elevenin analogs without the disulfide bond could not activate any of the three receptors (Fig. 8), supporting that the mature elevenin in *Aplysia* likely forms a disulfide bond and the disulfide bond is important for receptor activity. We recently identified another *Aplysia* peptide with a disulfide bond, i.e., allatostatin-C (SHYSSMCMFNVVACY)^8^. In this case, *Aplysia* AstC without a disulfide bond could activate its receptor: AstC-R, although with a significantly higher EC_50_ value compared to that in response to AstC with the disulfide bond. It is notable that there are 6 residues between the two cysteines in AstC, compared to 8 in elevenin. Perhaps, the smaller number of residues between the two cysteines allowed some of the AstC to form a disulfide bond spontaneously so that AstC without the disulfide bond could activate AstC-R at a lower potency. For comparison, for the *Aplysia* vasotocin (a homolog of mammalian vasopressin/oxytocin) with a disulfide bond (CFIRNCPKG-amide, 4 residues between the two cysteines) we characterized recently^62^, the analog without the disulfide bond actually had similar EC_50_ values on the two vasotocin receptors to the vasotocin with the disulfide bond. However, when the cysteines are protected by acetamidomethy to prevent the possible spontaneous formation of the disulfide bond, the receptor activity was dramatically reduced, supporting the importance of the vasotocin disulfide bond for receptor activity.

Our alanine substitution experiments demonstrated the specific roles of conserved residues in elevenin for receptor activation. We selected seven residues that are the most conserved ones except the two cysteines, i.e., G17, R16, P13, D5, F11, V18, and I4 with their degree of conserveness in this order (Fig. 1). Indeed, for apElevR1 and apElevR3, the substitution of G17 with alanine had the largest effect, and substitution of I4 and V18 had lowest effects (Fig. 9, Table 2), as also demonstrated for other neuropeptides^61^. Interestingly, for apElevR2, although substitution of G17 with alanine tended to have the largest effect, the substitution of the other five residues also had similarly profound effects, indicating apElevR2 was more sensitive to residue substitution compared to apElevR1 and apElevR3. For comparison, in several other peptides with the disulfide bond, alanine substitution of residues within the disulfide bond tends to have a larger impact on receptor activity than those outside the disulfide bond^62,68,69^. Thus, different receptors might have different sensitivities to alanine substitution in peptides with a disulfide bond.

Given that the most conserved residues are located between D5 and G17, we also made a truncated elevenin analog (elevenin_5-17_) that included only these 13 residues, and another one (elevenin_6-15_) that only included the two cysteines and the 8 residues between them (a total of 10 residues). If the elevenin activity depended primarily on the disulfide bond and the conserved residues, we would expect a small impact of elevenin_5-17_ on receptor activity. However, this is not what we observed (Fig. 10), indicating that the other residues and/or the overall peptide structure maintained by the complete 20 residues also played some roles in receptor activation. We did find that the shorter elevenin_6-15_ had a bigger impact than elevenin_5-17_, which is expected because there are fewer conserved residues in elevenin_6-15_ (Fig. 10). Again, apElevR2 was more sensitive to residue deletion compared to apElevR1 and apElevR3. Overall, both alanine substitution and residue deletion experiments indicated that even though the disulfide bond is essential, conserved residues, other less conserved residues, and/or the overall structure of the peptide might also play roles in receptor activation. Moreover, apElevR2 is more sensitive to alternation of the elevenin ligand than apElevR1 and apElevR3. Along this line, it is worth noting that apElevR2 also differed from apElevR1 and apElevR3 in that it had relatively low maximum IP1 responses (Fig. 8). Future work is needed to determine how the elevenin and its analogs interact with these receptors, perhaps using the approach involving AI protein structure prediction-based modeling described recently^61^. Such studies may inform us how and why the three receptors might have different sensitivities to alanine substitutions and residue deletion of the elevenin.

In summary, we have identified three elevenin receptors in *Aplysia*, which, together with the elevenin precursor, constitute a complete characterization of the elevenin signaling system in a mollusc. Given that several neural circuits that mediate a variety of behaviors, such as feeding and locomotion^34,36,40,70^, have been studied in *Aplysia*, it would be of interest to determine whether elevenin might play a modulatory role in these circuits. Indeed, RNA-seq data of different tissues (Supplementary Fig. 3) showed that the elevenin precursor is most highly expressed in the CNS, whereas apElevR1 and apElevR2 are not expressed most highly in the CNS, but they are present in the CNS. These data suggest that the elevenin signaling system might play some roles in these and/or other neural circuits. In addition, apElevR1 and apElevR2 are also highly expressed in the statocyst, and/or the mantle and gills, suggesting that the elevenin signaling system might also play roles in equilibrium reception (statocyst) or respiratory (the mantle and gills) functions in *Aplysia*^71^. It is expected that future studies will clarify these issues and provide a better understanding of the functions of the elevenin signaling system in protostomes. Finally, as elevenin-like peptides are present in cone snail venoms^66,67^, these studies might also provide insights that help promote the development of drugs.

## Material and methods

### Subjects and reagents

*Aplysia californica* (100–350 g) was obtained from Marinus, California, USA. *Aplysia* are hermaphroditic (i.e., each animal has reproductive organs normally associated with both male and female sexes). Animals were maintained by circulating artificial seawater at 14–16 °C and the animal room was set with a 24 h light cycle with a light period from 6:00 am to 6:00 pm. All chemicals were purchased from Sigma-Aldrich unless otherwise stated.

### Bioinformatic analysis of elevenin receptors

We first used NCBI to search for specific receptors and selected the nucleic acid sequences of receptors from species evolutionarily close to *Aplysia*, e.g., *Platynereis*. We performed BLASTn in NCBI and selected possible elevenin receptors according to E-value and similarity (positive). These sequences found by BLASTn were also searched in the AplysiaTools (http://www.aplysiatools.org/) to obtain more similar sequences for comparison. The complete nucleic acid sequences were searched for open reading frames (ORFs) in NCBI (https://www.ncbi.nlm.nih.gov/orffinder/). Then the amino acid sequences of the putative receptors were subsequently analyzed in TMHMM-2.0 (https://dtu.biolib.com/DeepTMHMM) to determine whether they are a seven-transmembrane GPCR receptor. After the putative receptors were identified, phylogenetic trees were constructed using ClustalW alignment and the maximum likelihood method of 1000 replicates in MEGA X (https://www.megasoftware.net/). Besides, we used the LG + G + F model to run all the phylogenetic trees.

### RNA extraction

The ganglia were removed from *Aplysia*, and Trizol was added to make up to 1 ml. The tissue was crushed by sonication, then, 200 μl chloroform was added. The solution was kept on ice for 15 min and was then centrifuged (10,000 × g, 4 °C, 15 min). The supernatant was transferred into a new centrifuge tube. An equal volume of pre-cooled isopropanol was added to the tube on ice. After standing at -20 °C overnight, the tube was centrifuged (10,000 × g, 4 °C, 10 min), and the supernatant was discarded. The pellet was washed twice with 1 ml of 75% ethanol/water and the centrifuge tube was shaken gently by hand to suspend the pellet. The tube was centrifuged again (12,000 × g, 4 °C, 10 min), the supernatant was discarded, and the precipitant was dried at room temperature for 5–10 min. Finally, 20 μl of nuclease-free water was added to dissolve the RNA and the concentration of RNA was measured.

### Reverse transcription and PCR

RNA was reverse transcribed into cDNA using the reverse transcription kit (Takara, RR036A) according to the instructions. The cDNA obtained after reverse transcription was used as a template for PCR. Based on the nucleic acid sequence of the putative elevenin receptor, each pair of specific primers (see Supplementary Table 2) was designed in NCBI. We cloned the elevenin precursor and the three elevenin receptors. Both PCR reactions were performed at 98 °C/2 min pre-denaturation, 98 °C/10 s denaturation, ~ 60 °C (depending on specific primers: see Supplementary Table 2)/15 s annealing, 72 °C/30 s extension (depending on specific PCR products), and 72 °C/5 min for another 35 cycles. After running PCR, double digestion, ligation, plasmid transfection, plate plating, and single clone picking, the receptor was cloned into the vector pcDNA3.1( +). Then the elevenin receptor with pcDNA3.1( +) was sequenced and aligned to ensure the sequence is correct.

### IP1 accumulation assay

The metabolic inositol phosphate cascade is usually caused by the regulation of phospholipase C-β (PLC-β) associated with the Gαq subunit of heterotrimeric G proteins. Gαq-coupled GPCRs stimulate PLC-β activity, resulting in increased cellular D-Myo-Inositol 1-Phosphate (IP1). When the G protein-coupled receptor (GPCR) expressed in CHO-K1 cells (Procell, CL-0062) is activated, it is generated by the Gαq pathway through the appropriate ligand, and IP3 is formed which is degraded to IP1. Therefore, IP1 can be used as an alternative to IP3. First, the putative receptor cloned into the mammalian expression vector pcDNA3.1( +) of *Aplysia* was transiently expressed in CHO-K1 cells. CHO-K1 cells were cultured in F-12 K medium (Gibco, 21,127–022) containing 10% fetal bovine serum (Genial, G11-70,500) at 37 °C and 5% CO_2_. When the cell density in the 6-well plate reached 70–90%, 1.5 μg of putative receptor plasmid [in pcDNA3.1( +)] was mixed with 200 μl buffer (Polyplus) in a 1.5 ml EP tube and incubated at room temperature for 10 min. The DNA/Transfection reagent (Polyplus) mixture was then added dropwise to the 6-well plate, mixed gently, and the cells were incubated overnight at 37 °C and 5% CO_2_. The next day, cells were digested, counted, diluted to a density of 40,0000 cells/ml, and replated in a white 384-well assay plate (Greiner bio-one, B14061RI), each well inoculated with the diluted liquid 50 μl and incubate overnight at 37 °C in 5% CO_2_. On the third day, the CHO cells were incubated for 1 h after adding peptide. The activation of putative receptors was detected by monitoring IP1 accumulation using the IP1 detection kit in Tecan Spark (Cisbio, 62IPAPEB). All procedures were performed according to the manufacturer's instructions for the IP1 assay kit. The elevenin peptide and analogs were synthesized by Sangon Biotech or Guoping Pharmaceutical (Supplementary Fig. 4) and were aliquoted in 50 nmol EP tubes and stored at -20 °C until use.

### Statistical analysis

Dose–response curves and bar graphs for experimental data were plotted using Prism software (GraphPad). Data are expressed as the mean ± SEM. Final EC_50_ values are rounded to two significant figures. All experimental data were taken from individual animals or preparations, and n refers to the number of preparations. Data were considered significant when *P* < 0.05. Statistical tests were performed using Prism software. They included Student’s t-test (two groups) and one-way ANOVA (three or more groups). When one-way ANOVA shows a significant difference, individual comparisons with Bonferroni corrections were then performed.

## Supplementary Information

Supplementary Table 1.

Supplementary Table 2.

Supplementary Table 3.

Supplementary Table 4.

Supplementary Figures.

Supplementary Figure 4.

## Data Availability

The datasets used and/or analyzed during the current study are available from the corresponding author upon reasonable request.
