# Supplementary material for: Identification of three elevenin receptors and roles of elevenin disulfide bond and residues in receptor activation in Aplysia californica
Source: Sci Rep. 2023 May 11;13:7662. doi: 10.1038/s41598-023-34596-9 (PMC10175484; doi:10.1038/s41598-023-34596-9)
Supplement: Supplementary file 5 — Supplementary Figures. [file 41598_2023_34596_MOESM5_ESM.docx]

Supplement to: **Identification of three elevenin receptors and roles of elevenin disulfide bond and residues in receptor activation in** ***Aplysia californica***

Ping Fu^1^, Yu-Shuo Mei^1^, Wei‑Jia Liu^1^, Ping Chen^1^, Qing-Chun Jin^1^, Shi‑Qi Guo^1^, Hui‑Ying Wang^1^, Ju‑Ping Xu^1^, Yan-chu-fei Zhang^1^, Xue‑Ying Ding^1^, Cui-Ping Liu^1^, Cheng-Yi Liu^1^, Rui-Ting Mao^1^, Guo Zhang^1^* & Jian Jing ^1,2,3^*

Supplementary information includes 4 supplementary tables (as separate Excel files), and 4 supplemental figures (3 in this file, Supplementary Fig. 4 is a separate PDF file).

**
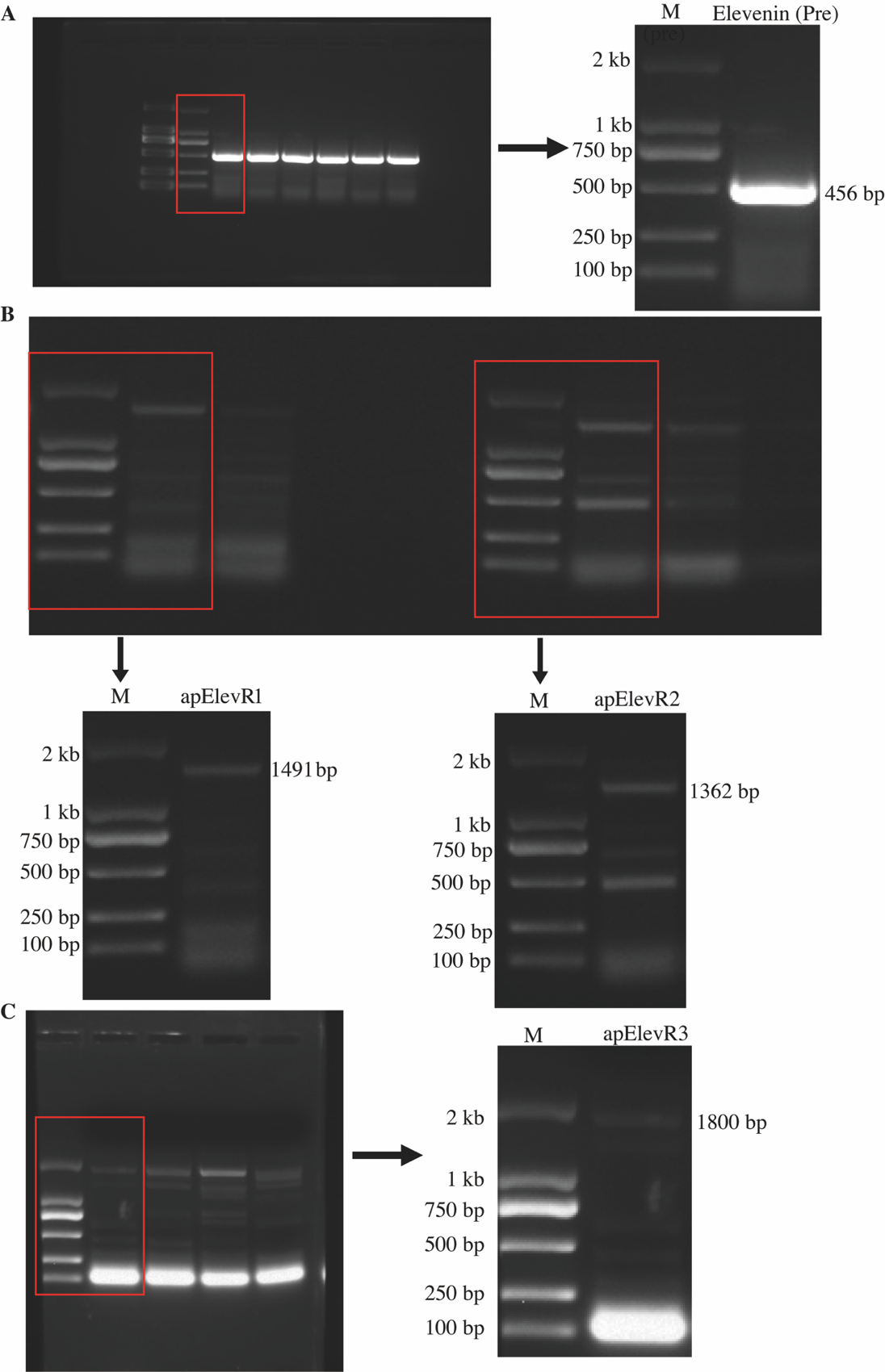
**

**Supplementary Figure 1.** **Complete gels for PCR experiments.** (**A**) Left panels: complete gels of the elevenin precursor and all six right lanes are from abdominal ganglion cDNA; Right panels, the corresponding cropped gel shown in Fig. 2A. (**B**) upper panels: complete gels of elevenin R1 and R2; Lower panels, corresponding cropped gel shown in Fig. 2B-C. (**C**) Left panels: the complete gel of elevenin R3; Right panels, the corresponding cropped gel shown in Fig. 2D; (The images in the red box are shown in Fig. 2)

**
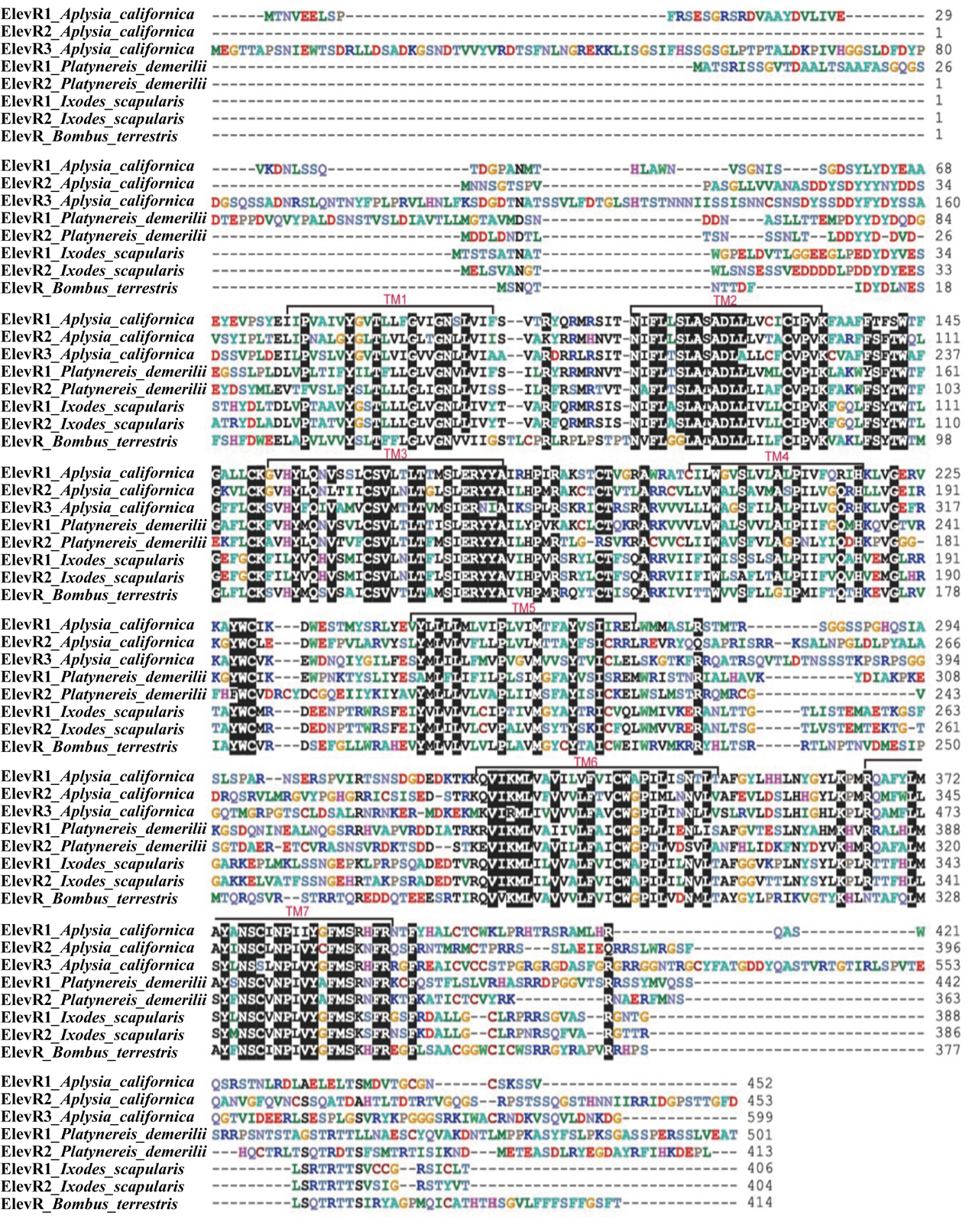
**

**Supplementary Figure 2. Comparison of elevenin receptors in *Aplysia* with sequences in an annelid (*Platynereis dumerilii*) and two arthropods (*****Ixodes scapularis* and *Bombus terrestris*).** The alignment is performed using BioEdit v5.0.6 (ClustalW Multiple alignment - Graphic View). Predicted 7 transmembranes (TM) domains.


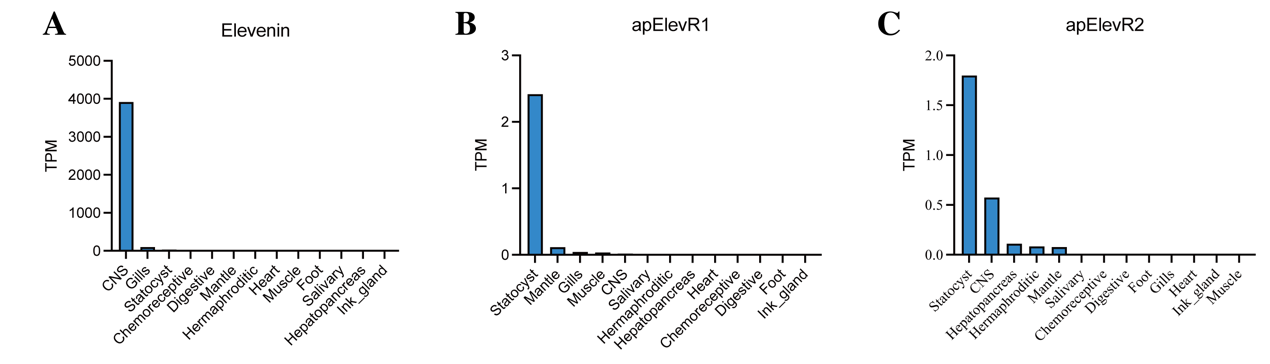


**Supplementary Figure 3**. **The expression of elevenin precursor and its receptors in the CNS and peripheral tissues of Aplysia.** (**A**) Quantification of the expression of elevenin precursor; (**B**) Quantification of the expression of apElevR1; (**C**) Quantification of the expression of apElevR2. The RNA-seq data is represented as TPM (transcript per million) values. See Text for details

**Supplementary Figure 4.** Peptide synthesis and quality information from commercial companies. See Supplementary Fig4.pdf.
