# Supplementary material for: Identification of three elevenin receptors and roles of elevenin disulfide bond and residues in receptor activation in Aplysia californica
Source: Sci Rep. 2023 May 11;13:7662. doi: 10.1038/s41598-023-34596-9 (PMC10175484; doi:10.1038/s41598-023-34596-9)
Supplement: Supplementary file 6 — Supplementary Figure 4. [file 41598_2023_34596_MOESM6_ESM.pdf]

Contents

Elevenin_COA **1**

Elevenin_HPLC 2

Elevenin_LCMS 3

Elevenin'_COA **4**

Elevenin'_HPLC 5

Elevenin'_LCMS 6

[Ala^{4}]Elevenin_COA **7**

[Ala^{4}]Elevenin_HPLC 8

[Ala^{4}]Elevenin_LCMS 9

[Ala^{5}]Elevenin_COA **10**

[Ala^{5}]Elevenin_HPLC 11

[Ala^{5}]Elevenin_LCMS 12

[Ala^{11}]Elevenin_COA **13**

[Ala^{11}]Elevenin_HPLC 14

[Ala^{11}]Elevenin_LCMS 15

[Ala^{13}]Elevenin_COA **16**

[Ala^{13}]Elevenin_HPLC 17

[Ala^{13}]Elevenin_LCMS 18

[Ala^{16}]Elevenin_COA **19**

[Ala^{16}]Elevenin_HPLC 20

[Ala^{16}]Elevenin_LCMS 21

[Ala^{17}]Elevenin_COA **22**

[Ala^{17}]Elevenin_HPLC 23

[Ala^{17}]Elevenin_LCMS 24

[Ala^{18}]Elevenin_COA **25**

[Ala^{18}]Elevenin_HPLC 26

[Ala^{18}]Elevenin_LCMS 27

Elevenin$_{5-17}$_COA **28**

Elevenin$_{5-17}$_HPLC 29

Elevenin$_{5-17}$_LCMS 30

Elevenin$_{6-15}$_COA **31**

Elevenin$_{6-15}$_HPLC 32

Elevenin$_{6-15}$_LCMS 33

Elevenin_COA

Sangon Biotech 多肽合成报告单

CERTIFICATE OF ANALYSIS

**Product Name**	P23911
**Catalog No.**	N/A
**Lot No.**	P23911-20211021
**Sequence**	RPRIDCTRFVFAPACRGVSA
**Length**	20AA
**Modification**	C-C
**Molecular Weight (MW)**	2220.62
**Storage**	-20°C

**Test Items**	**Specifications**	**Results**
**MW by MS**	2220.2	Conforms
**Purity by HPLC**	> 95%	98.831%
**Peptide Content**	N/A	N/A
**Acetic acid content**	N/A	N/A
**Appearance**	White to off-white lyophilized powde	Conforms
**Quantity**	2mg*5vials	10.0mg

Certified by: Melinda

Date 11/05/2021

Quality Assurance Department

Elevenin_HPLC

生工® Sangon Biotech

Sample Information

Name : P23911
Sequence : RPRIDCTRFVFAPACRGVSA
Modification : C-C
Lot.No : P23911-20211021
Pump A : 0.1%trifluoroacetic in 100water
Pump B : 0.1%trifluoroacetic in 100%acetonrtrile
Total Flow : 1.0ml/min
Wavelength : 214nm
Analytical column type : NanoChrom Chromcore TM120 C18(4.6*250MM*5UM)
Dissolution method : 0.1mg sample dissolved to 0.5mL by 100%H2O
Acquisition Time : 2021/11/05 12:34:22
Inj.Volume : 40ul

Time	Module	Action	Value
0.01	Pumps	B.Conc	24
20.00	Pumps	B.Conc	44

Chromatogram

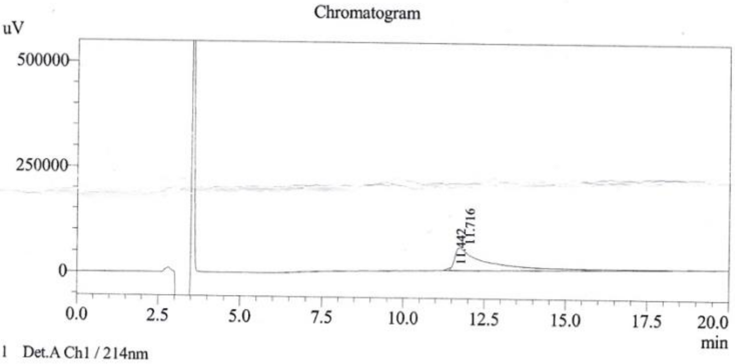

1 Det.A Ch1 / 214nm

PeakTable

Detector A Ch1 214nm

Peak#	Ret. Time	Area	Height	Area %	Height %
1	11.442	44706	5877	1.169	9.981
2	11.716	3780707	53007	98.831	90.019
Total		3825413	58883	100.000	100.000

Elevenin_LCMS

Sangon Biotech

MS Spectrum

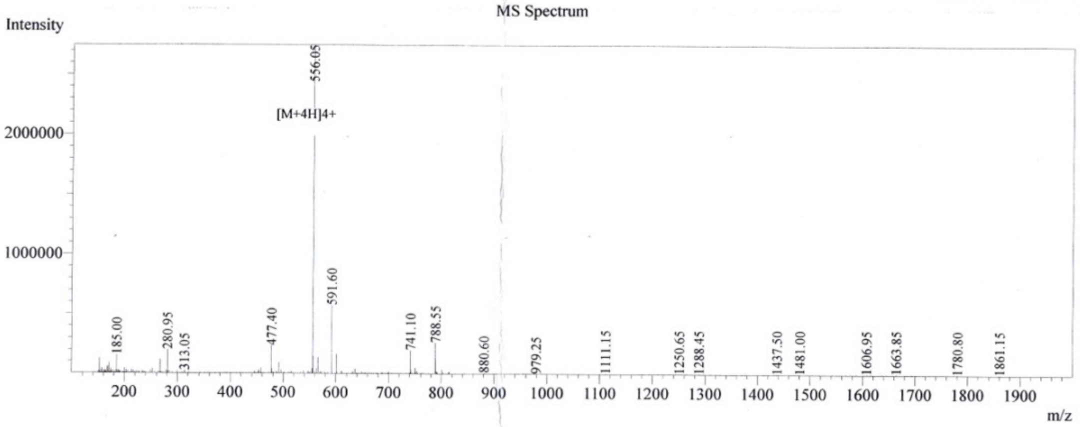

Sample Information
Dissolution method :0.1mg sample dissolved to 0.5mL by 100%H2O
Date Acquired :2021/11/05 12:39:26
Injection Volume :1ul

Interface :ESI
Nebulizing Gas Flow :1.50L/min
CDL Temp :250°C
CDL Volt :0v
Block Temp :200

Prerod Bias :+4.5kv
Detector :-0.2kv
T.Flow :0.2ml/min
B.conc :50%H2O/50%MeOH

Name :P23911
Sequence :RPRIDCTRFVFAPACRGVSA
Modification :C-C
Lot No. :P23911-20211021
Theoretical :2220.62
bserved :2220.20

生工生物工程（上海）股份有限公司

地址：上海市松江区香闵路698号
电话/Tel: 400-821-0268
邮箱/Email: Sales@sangon.com

Add: 698 Xiang Min Road Songjiang Shanghai China
传真/Fax: 86-21-37772170
网址/Web: wwww.sangon.com

Elevenin'_COA

国平药业
GUOPING PHARMACEUTICAL

安徽省国平药业有限公司

CERTIFICATE OF ANALYSIS

**Order ID**	GP120586
**Name**	N/A
**Lot No.**	GP120586-0310
**Sequence**	RPRIDCTRFVFAPACRGVSA
**Dissolution condition**	100%H2O
**Length**	20AA
**Modification**	N/A
**Molecular Weight (MW)**	2222.59
**Storage**	-20°C

**Test Items**	**Specifications**	**Results**
**MW by MS**	2222.20	Conforms
**Purity by HPLC**	>95%	95.145%
**Peptide Content**	N/A	N/A
**Moisture content**	N/A	N/A
**Acetic acid content**	N/A	N/A
**Appearance**	White to off-white lyophilized powder	Conforms
**Quantity**	10mg	10.0mg

Certified by: LiuHui　　Date　03/28/2022

Quality Assurance Department

Note: this product is intended for research use only; not for diagnostic or human use.

Guoping Pharmaceutical Co., LTD

地址:合肥市经开区桃花工业园拓展区工投立恒工业广场A2西F1,电话:0551-62841987 传真:0551-62841765 www.guopingyaoye.com

Elevenin'_HPLC

国平药业
GUOPING PHARMACEUTICAL

Sample Information

Order ID	:GP120586
Name	:N/A
Sequence	:RPRIDCTRFVFAPACRGVSA
Lot.No	:GP120586-0310
Pump A	:0.1%Trifluoroacetic in 100% water
Pump B	:0.1%Trifluoroacetic in 100% acetonitrile
Total Flow	:1ml/min
Wavelength	:220nm
Analytical column type	:SHIMADZU Inertsil ODS-SP(4.6*250mm*5um)
Dissolution method	:100%H2O
Inj. Volume	:12 uL

Time	Module	Action	Value
0.01	Pumps	B.Conc	15
20.00	Pumps	B.Conc	55
23.00	Pumps	B.Conc	100
38.00	Pumps	B.Conc	100
40.00	Pumps	B.Conc	15
50.00	Controller	Stop	

Chromatogram

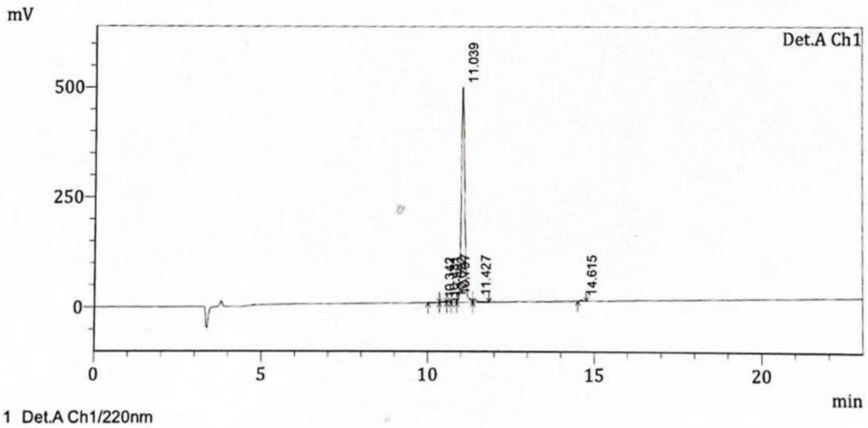

1 Det.A Ch1/220nm

PeakTable

Detector A Ch1 220nm

Peak#	Ret. Time	Area	Height	Area %	Height %
1	10.342	6587	471	0.177	0.092
2	10.481	23091	2760	0.620	0.538
3	10.692	20432	3132	0.548	0.610
4	10.797	62613	7719	1.681	1.504
5	11.039	3544286	490832	95.145	95.662
6	11.427	48632	5191	1.306	1.012
7	14.615	19492	2984	0.523	0.582
Total		3725134	513089	100.000	100.000

Elevenin'_LCMS

国平药业
GUOPING PHARMACEUTICAL

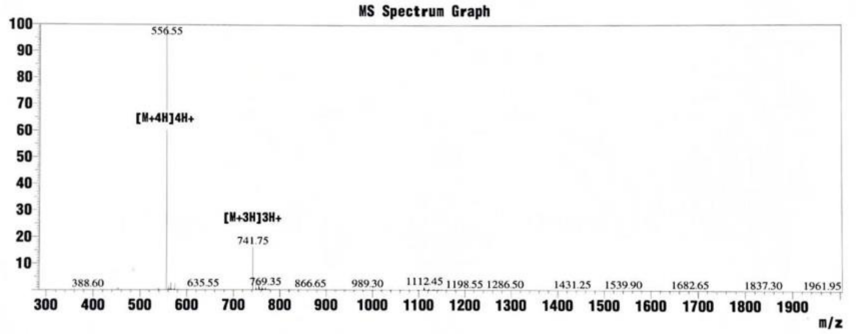

Sample Information

Dissolution method	:5%HAC+8%ACN+87%H20	Interface	:ESI	Prerod Bias	:+1.5kv
Modified Date	:2022/03/28	Nebulizing Gas Flow	:1.50L/min	Detector	:-0.2kv
Injection Volume	:1ul	CDL Temp	:250C	T.Flow	:0.2ml/min
Heat Block Temp	:200	CDL Volt	:0v	B.conc	:50%H20/50%MEOH

Order ID	:GP120586
Name	:N/A
Sequence	:RPRIDCTRFVFAPACRGVSA
Lot.No	:GP120586-0310
Theoretical	:2222.59
Observed	:2222.20

[Ala^4]Elevenin_COA

Sangon Biotech

多肽合成报告单

CERTIFICATE OF ANALYSIS

**Product Name**	P27392-1
**Catalog No.**	N/A
**Lot No.**	P27392-1-23021501
**Sequence**	RPRADCTRFVFAPACRGVSA
**Length**	20AA
**Modification**	C-C
**Molecular Weight (MW)**	2178.54
**Storage**	-20°C

Test Items	Specifications	Results
MW by MS	2178.3	Conforms
Purity by HPLC	> 95%	97.878%
Peptide Content	N/A	N/A
Acetic acid content	N/A	N/A
Appearance	White to off-white lyophilized powde	Conforms
Quantity	1mg*5vials	5.0mg

Certified by: Melinda

Date 02/28/2023

Quality Assurance Department

[Ala4]Elevenin_HPLC

Sangon Biotech

Sample Information

Name	:	P27392-1
Sequence	:	RPRADCTRFVFAPACRGVSA
Modification	:	C-C
Lot.No	:	P27392-1-23021501
Pump A	:	0.1%trifluoroacetic in 100%water
Pump B	:	0.1%trifluoroacetic in 100%acetonrtrile
Total Flow	:	1.0ml/min
Wavelength	:	214nm
Analytical column type	:	SHIMADZU shim-pack GIST(4.6*250MM*5UM)
Dissolution method	:	0.1mg sample dissolved to 0.5mL by 10%ACN and 90%H2O
Acquisition Time	:	2023/02/28 09:27:36
Inj.Volume	:	30ul

Time	Module	Action	Value
0.01	Pumps	B.Conc	23
20.00	Pumps	B.Conc	43

Chromatogram

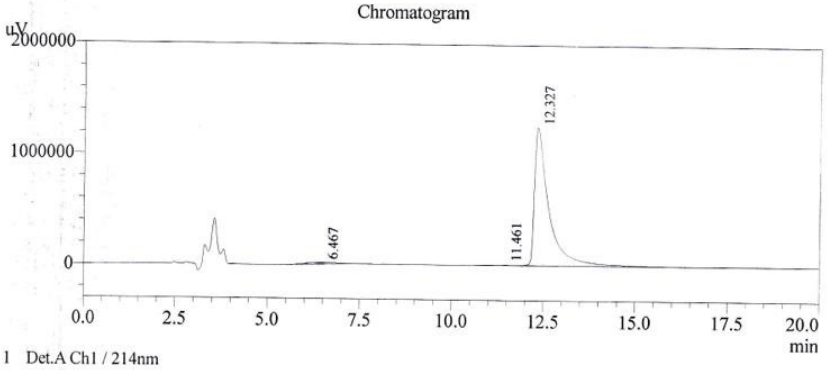

1 Det.A Ch1 / 214nm

PeakTable

Detector A Ch1 214nm

Peak#	Ret. Time	Area	Height	Area %	Height %
1	6.467	693043	11987	1.972	0.950
2	11.461	52740	5517	0.150	0.437
3	12.327	34405930	1243729	97.878	98.612
Total		35151714	1261233	100.000	100.000

生工生物工程（上海）股份有限公司

地址: 上海市松江区香闵路698号
电话/Tel: 400-821-0268
邮箱/Email: sales@sangon.com

Add: 698 Xiang Min Road SongJiang Shanghai China
传真/Fax: 86-21-37772170
网址/Web: www.sangon.com

[Ala^{4}]Elevenin__LCMS

Sangon Biotech

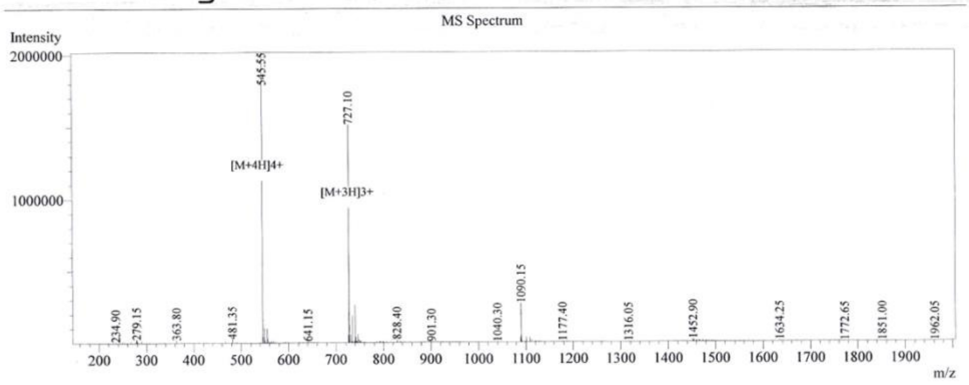

Sample Information		Interface	:ESI	Prerod Bias	:+4.5kv
Dissolution method	:0.1mg sample dissolved to 0.5mL by 50%ACN and 50%H2O	Nebulizing Gas Flow	:1.50L/min	Detector	:-0.2kv
		CDL Temp	:250℃	T.Flow	:0.2ml/min
Date Acquired	:2023/02/28 10:04:31	CDL Volt	:0v	B.conc	:50%H2O/50%MeOH
Injection Volume	:1ul	Block Temp	:200		

Name	:P27392-1
Sequence	:RPRADCTRFVFAPACRGVSA
Modification	:C-C
Lot No.	:P27392-1-23021501
Theoretical	:2178.536
Observed	:2178.30

生工生物工程（上海）股份有限公司

地址：上海市松江区香闵路698号
Add: 698 Xiang Min Road Songjiang Shanghai China
电话/Tel: 400-821-0268
传真/Fax: 86-21-37772170
邮箱/Email: Sales@sangon.com
网址/Web: wwww.sangon.com

[Ala^5]Elevenin_COA

Sangon Biotech 多肽合成报告单

CERTIFICATE OF ANALYSIS

Product Name	P25315-4
Catalog No.	N/A
Lot No.	P25315-4-220506
Sequence	RPRIACTRFVFAPACRGVSA
Length	20AA
Modification	C-C
Molecular Weight (MW)	2176.61
Storage	-20°C

Test Items	Specifications	Results
MW by MS	2176.2	Conforms
Purity by HPLC	> 98%	98.553%
Peptide Content	N/A	N/A
Acetic acid content	N/A	N/A
Appearance	White to off-white lyophilized powde	Conforms
Quantity	1mg*5vials	5.0mg

Certified by: Melinda

Date 05/20/2022

Quality Assurance Department

[Ala^5]Elevenin_HPLC

Sangon Biotech

Sample Information

Name	:	P25315-4
Sequence	:	RPRIACTRFVFAPACRGVSA
Modification	:	C-C
Lot.No	:	P25315-4-220506
Pump A	:	0.1%trifluoroacetic in 100%water
Pump B	:	0.1%trifluoroacetic in 100%acetonrtrile
Total Flow	:	1.0ml/min
Wavelength	:	214nm
Analytical column type	:	SHIMADZU Inertsil ODS-SP(4.6*250MM*5UM)
Dissolution method	:	0.1mg sample dissolved to 0.5mL by 20%ACN and 80%H2O
Acquisition Time	:	2022/05/20 14:33:23
Inj.Volume	:	60ul

Time	Module	Action	Value
0.01	Pumps	B.Conc	25
20.00	Pumps	B.Conc	45

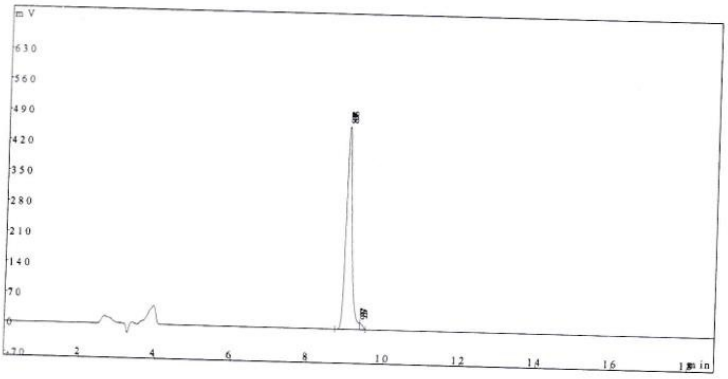

PeakTable

Detector A Ch 214nm

Peak#	Ret.Time	Area	Height	Area%	Height%
1	8.998	4838502	466373	98.553	96.846
2	9.357	71052	15189	1.447	3.154
Total		4909554	481562	100.000	100.000

生工生物工程（上海）股份有限公司

地址: 上海市松江区香闵路698号
电话/Tel: 400-821-0268
邮箱/Email: sales@sangon.com

Add: 698 Xiang Min Road SongJiang Shanghai China
传真/Fax: 86-21-37772170
网址/Web: www.sangon.com

[Ala^5]Elevenin_LCMS

Sangon Biotech

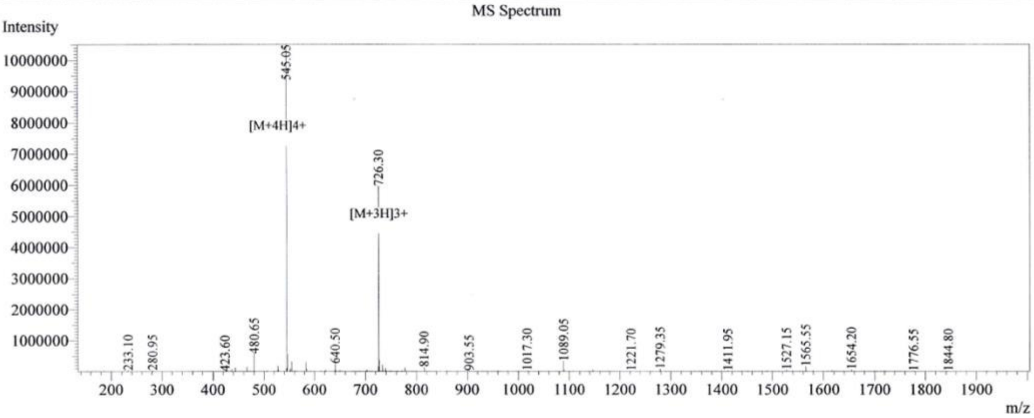

Sample Information
Dissolution method :0.1mg sample dissolved to 0.5mL by 50%ACN and 50%H2O
Date Acquired :2022/05/20 14:38:10
Injection Volume :1ul

Interface :ESI
Nebulizing Gas Flow :1.50L/min
CDL Temp :250℃
CDL Volt :0v
Block Temp :200

Prerod Bias :+4.5kv
Detector :-0.2kv
T.Flow :0.2ml/min
B.conc :50%H2O/50%MeOH

Name :P25315-4
Sequence :RPRIACTRFVFAPACRGVSA
Modification :C-C
Lot No. :P25315-4-220506
Theoretical :2176.607
bserved :2176.20

生工生物工程（上海）股份有限公司

地址：上海市松江区香闵路698号
电话/Tel: 400-821-0268
邮箱/Email: Sales@sangon.com

Add: 698 Xiang Min Road Songjiang Shanghai China
传真/Fax: 86-21-37772170
网址/Web: wwww.sangon.com

[Ala^{11}]Elevenin_COA

Sangon Biotech　　　　多肽合成报告单

CERTIFICATE OF ANALYSIS

Product Name	P27392-2
Catalog No.	N/A
Lot No.	P27392-2-23021501
Sequence	RPRIDCTRFVAAPACRGVSA
Length	20AA
Modification	C-C
Molecular Weight (MW)	2144.52
Storage	-20°C

Test Items	Specifications	Results
MW by MS	2144.4	Conforms
Purity by HPLC	> 95%	96.968%
Peptide Content	N/A	N/A
Acetic acid content	N/A	N/A
Appearance	White to off-white lyophilized powde	Conforms
Quantity	1mg*5vials	5.0mg

Certified by: Melinda　　Date 02/28/2023

Quality Assurance Department

[Ala^{11}]Elevenin_HPLS

Sangon Biotech

Sample Information

Name	: P27392-2		
Sequence	: RPRIDCTRFVAAPACRGVSA		
Modification	: C-C		
Lot.No	: P27392-2-23021501		
Pump A	: 0.1%trifluoroacetic in 100%water		
Pump B	: 0.1%trifluoroacetic in 100%acetonrtrile		
Total Flow	: 1.0ml/min		
Wavelength	: 214nm		
Analytical column type	: SHIMADZU Inertsil ODS-SP(4.6*250MM*5UM)		
Dissolution method	: 0.1mg sample dissolved to 0.5mL by 10%ACN and 90%H2O		
Acquisition Time	: 2023/02/28 13:15:39		
Inj.Volume	: 10ul		

Time	Module	Action	Value
0.01	Pumps	B.Conc	19
20.00	Pumps	B.Conc	39

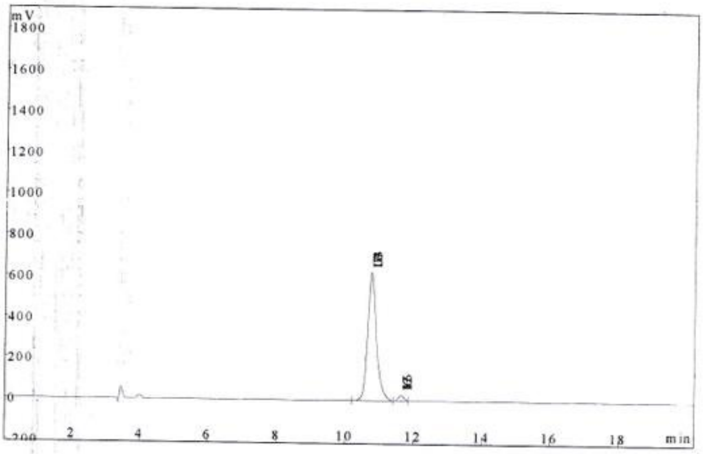

PeakTable

Detector A Ch 214nm

Peak#	Ret.Time	Area	Height	Area%	Height%
1	10.733	10763469	626569	96.968	95.854
2	11.626	336561	27100	3.032	4.146
Total		11100030	653669	100.000	100.000

生工生物工程（上海）股份有限公司

地址: 上海市松江区香闵路698号 /Add: 698 Xiang Min Road SongJiang Shanghai China
电话/Tel: 400-821-0268 传真/Fax: 86-21-37772170
邮箱/Email: sales@sangon.com 网址/Web: www.sangon.com

[Ala^{11}]Elevenin_ LCMS

Sangon Biotech

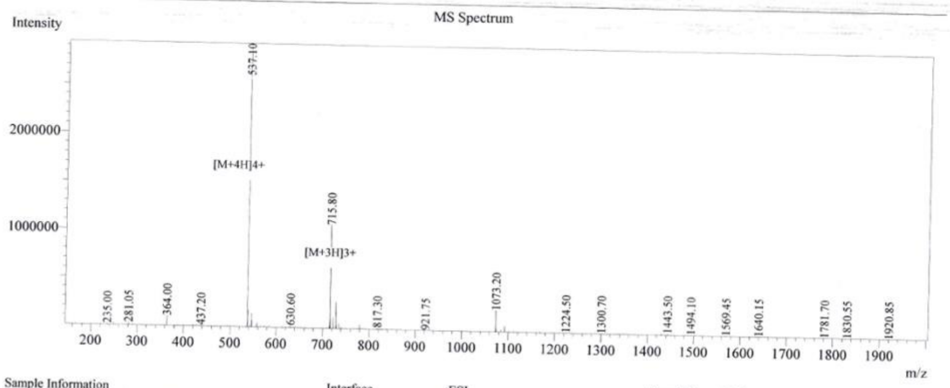

Sample Information
Dissolution method :0.1mg sample dissolved to 0.5mL by 50%ACN and 50%H2O
Date Acquired :2023/02/28 14:36:54
Injection Volume :1ul

Interface :ESI
Nebulizing Gas Flow :1.50L/min
CDL Temp :250℃
CDL Volt :0v
Block Temp :200

Prerod Bias :+4.5kv
Detector :-0.2kv
T.Flow :0.2ml/min
B.conc :50%H2O/50%MeOH

Name :P27392-2
Sequence :RPRIDCTRFVAAPACRGVSA
Modification :C-C
Lot No. :P27392-2-23021501
Theoretical :2144.519
Observed :2144.40

生工生物工程（上海）股份有限公司

地址：上海市松江区香闵路698号
电话/Tel: 400-821-0268
邮箱/Email: Sales@sangon.com

Add: 698 Xiang Min Road Songjiang Shanghai China
传真/Fax: 86-21-37772170
网址/Web: wwww.sangon.com

[Ala^{13}]Elevenin_ COA

Sangon Biotech

多肽合成报告单

CERTIFICATE OF ANALYSIS

**Product Name**	P25315-3
**Catalog No.**	N/A
**Lot No.**	P25315-3-220506
**Sequence**	RPRIDCTRFVFAAACRGVSA
**Length**	20AA
**Modification**	C-C
**Molecular Weight (MW)**	2194.58
**Storage**	-20°C

**Test Items**	**Specifications**	**Results**
**MW by MS**	2193.8	Conforms
**Purity by HPLC**	> 98%	98.169%
**Peptide Content**	N/A	N/A
**Acetic acid content**	N/A	N/A
**Appearance**	White to off-white lyophilized powde	Conforms
**Quantity**	1mg*5vials	5.0mg

Certified by: Melinda

Date 05/20/2022

Quality Assurance Department

[Ala^{13}]Elevenin_ HPLC

Sangon Biotech

Sample Information

Name	:	P25315-3
Sequence	:	RPRIDCTRFVFAAACRGVSA
Modification	:	C-C
Lot.No	:	P25315-3-220506
Pump A	:	0.1%trifluoroacetic in 100%water
Pump B	:	0.1%trifluoroacetic in 100%acetonrtrile
Total Flow	:	1.0ml/min
Wavelength	:	214nm
Analytical column type	:	SHIMADZU Inertsil ODS-SP(4.6*250MM*5UM)
Dissolution method	:	0.1mg sample dissolved to 0.5mL by 10%ACN and 90%H2O
Acquisition Time	:	2022/05/20 13:26:15
Inj.Volume	:	20ul

Time	Module	Action	Value
0.01	Pumps	B.Conc	23
20.00	Pumps	B.Conc	43

Chromatogram

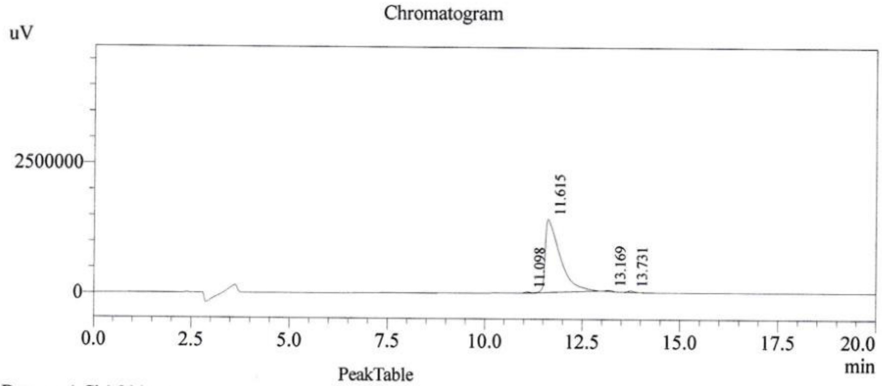

PeakTable

Detector A Ch1 214nm

Peak#	Ret. Time	Area	Height	Area %	Height %
1	11.098	176176	21777	0.460	1.470
2	11.615	37561236	1413925	98.169	95.465
3	13.169	179003	18558	0.468	1.253
4	13.731	345531	26825	0.903	1.811
Total		38261946	1481086	100.000	100.000

生工生物工程（上海）股份有限公司

地址: 上海市松江区香闵路698号
电话/Tel: 400-821-0268
邮箱/Email: sales@sangon.com

Add: 698 Xiang Min Road SongJiang Shanghai China
传真/Fax: 86-21-37772170
网址/Web: www.sangon.com

[Ala^{13}]Elevenin_ LCMS

Sangon Biotech

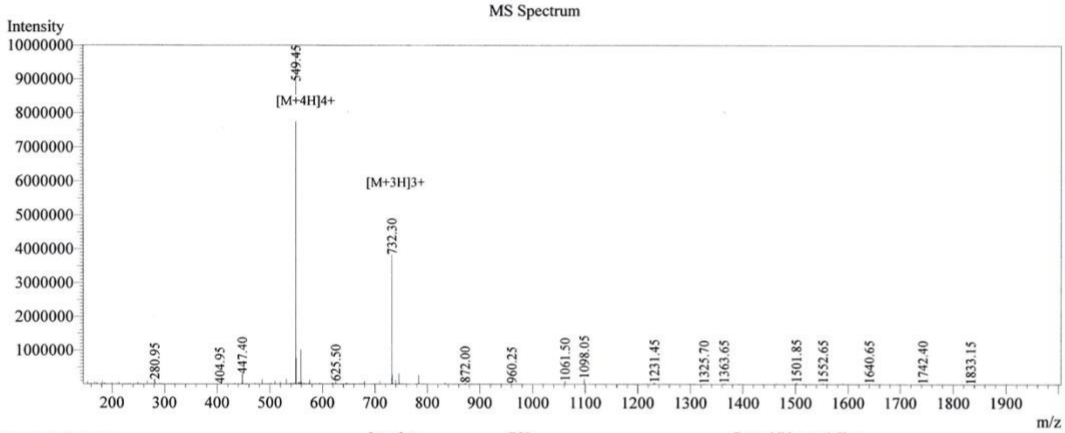

Sample Information
Dissolution method :0.1mg sample dissolved to 0.5mL by 50%ACN and 50%H2O
Date Acquired :2022/05/19 20:05:05
Injection Volume :1ul

Interface :ESI
Nebulizing Gas Flow :1.50L/min
CDL Temp :250℃
CDL Volt :0v
Block Temp :200

Prerod Bias :+4.5kv
Detector :-0.2kv
T.Flow :0.2ml/min
B.conc :50%H2O/50%MeOH

Name :P25315-3
Sequence :RPRIDCTRFVFAAACRGVSA
Modification :C-C
Lot No. :P25315-3-220506
Theoretical :2194.579
bserved :2193.80

生工生物工程（上海）股份有限公司

地址：上海市松江区香闵路698号
电话/Tel: 400-821-0268
邮箱/Email: Sales@sangon.com

Add: 698 Xiang Min Road Songjiang Shanghai China
传真/Fax: 86-21-37772170
网址/Web: wwww.sangon.com

[Ala^{16}]Elevenin_ COA

Sangon Biotech

多肽合成报告单

CERTIFICATE OF ANALYSIS

Product Name	P25315-2
Catalog No.	N/A
Lot No.	P25315-2-220506
Sequence	RPRIDCTRFVFAPACAGVSA
Length	20AA
Modification	C-C
Molecular Weight (MW)	2135.51
Storage	-20°C

Test Items	Specifications	Results
MW by MS	2134.7	Conforms
Purity by HPLC	> 98%	98.051%
Peptide Content	N/A	N/A
Acetic acid content	N/A	N/A
Appearance	White to off-white lyophilized powde	Conforms
Quantity	1mg*5vials	5.0mg

Certified by: Melinda

Date 05/20/2022

Quality Assurance Department

[Ala^{16}]Elevenin_ HPLC

Sangon Biotech

Sample Information

Name	:	P25315-2
Sequence	:	RPRIDCTRFVFAPACAGVSA
Modification	:	C-C
Lot.No	:	P25315-2-220506
Pump A	:	0.1%trifluoroacetic in 100%water
Pump B	:	0.1%trifluoroacetic in 100%acetonrtrile
Total Flow	:	1.0ml/min
Wavelength	:	214nm
Analytical column type	:	SHIMADZU Inertsil ODS-SP(4.6*250MM*5UM)
Dissolution method	:	0.1mg sample dissolved to 0.5mL by 10%ACN and 90%H2O
Acquisition Time	:	2022/05/20 12:21:44
Inj.Volume	:	20ul

Time	Module	Action	Value
0.01	Pumps	B.Conc	27
20.00	Pumps	B.Conc	47

Chromatogram

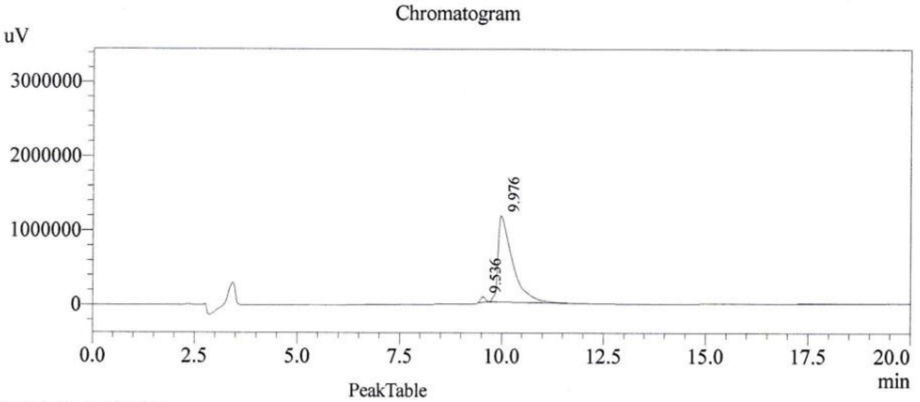

PeakTable

Detector A Ch1 214nm

Peak#	Ret. Time	Area	Height	Area %	Height %
1	9.536	576470	70833	1.949	5.749
2	9.976	29006669	1161290	98.051	94.251
Total		29583140	1232123	100.000	100.000

生工生物工程（上海）股份有限公司

地址: 上海市松江区香闵路698号
电话/Tel: 400-821-0268

Add: 698 Xiang Min Road SongJiang Shanghai China
传真/Fax: 86-21-37772170

[Ala^{16}]Elevenin_ LCMS

Sangon Biotech

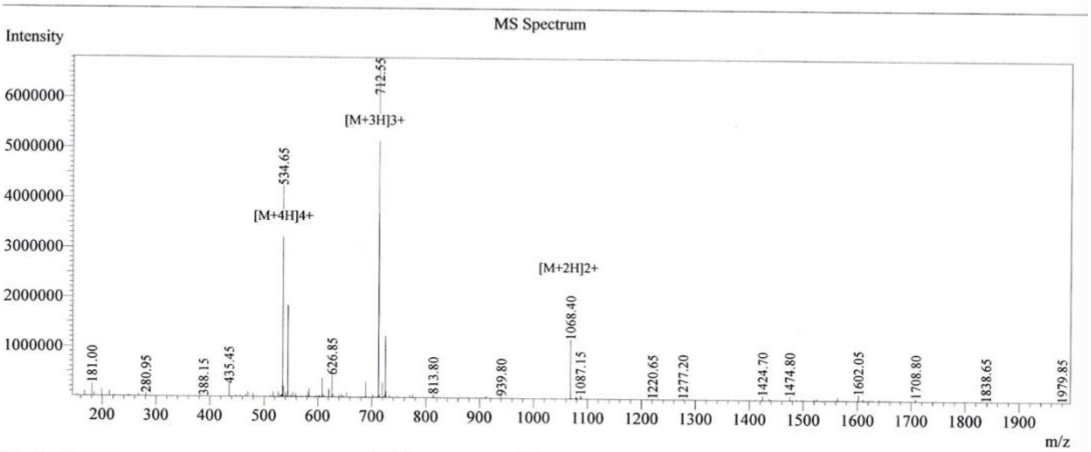

Sample Information
Dissolution method :0.1mg sample dissolved to 0.5mL by 50%ACN and 50%H2O
Date Acquired :2022/05/20 12:28:21
Injection Volume :1ul

Interface :ESI
Nebulizing Gas Flow :1.50L/min
CDL Temp :250℃
CDL Volt :0v
Block Temp :200

Prerod Bias :+4.5kv
Detector :-0.2kv
T.Flow :0.2ml/min
B.conc :50%H2O/50%MeOH

Name :P25315-2
Sequence :RPRIDCTRFVFAPACAGVSA
Modification :C-C
Lot No. :P25315-2-220506
Theoretical :2135.508
bserved :2134.65

生工生物工程（上海）股份有限公司

地址：上海市松江区香闵路698号
电话/Tel: 400-821-0268
邮箱/Email: Sales@sangon.com

Add: 698 Xiang Min Road Songjiang Shanghai China
传真/Fax: 86-21-37772170
网址/Web: wwww.sangon.com

[Ala^{17}]Elevenin _COA

Sangon Biotech 多肽合成报告单

CERTIFICATE OF ANALYSIS

Product Name	P25315-1
Catalog No.	N/A
Lot No.	P25315-1-220506
Sequence	RPRIDCTRFVFAPACRAVSA
Length	20AA
Modification	C-C
Molecular Weight (MW)	2234.64
Storage	-20°C

Test Items	Specifications	Results
MW by MS	2234.0	Conforms
Purity by HPLC	> 98%	98.221%
Peptide Content	N/A	N/A
Acetic acid content	N/A	N/A
Appearance	White to off-white lyophilized powde	Conforms
Quantity	1mg*5vials	5.0mg

Certified by: Melinda

Date 05/20/2022

Quality Assurance Department

[Ala[17]]Elevenin _HPLC

Sangon Biotech

Sample Information

Name	:	P25315-1
Sequence	:	RPRIDCTRFVFAPACRAVSA
Modification	:	C-C
Lot.No	:	P25315-1-220506
Pump A	:	0.1%trifluoroacetic in 100%water
Pump B	:	0.1%trifluoroacetic in 100%acetonrtrile
Total Flow	:	1.0ml/min
Wavelength	:	214nm
Analytical column type	:	SHIMADZU Inertsil ODS-SP(4.6*250MM*5UM)
Dissolution method	:	0.1mg sample dissolved to 0.5mL by 20%ACN and 80%H2O
Acquisition Time	:	2022/05/20 14:35:50
Inj.Volume	:	60ul

Time	Module	Action	Value
0.01	Pumps	B.Conc	25
20.00	Pumps	B.Conc	45

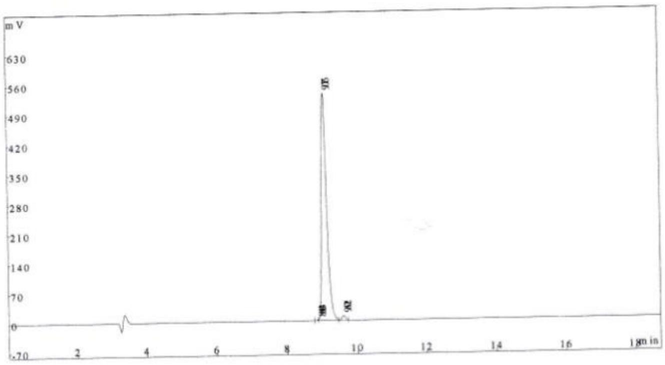

PeakTable

Detector A Ch 214nm

Peak#	Ret.Time	Area	Height	Area%	Height%
1	8.883	6867	2611	0.132	0.476
2	9.076	5085352	534150	98.221	97.378
3	9.602	85282	11770	1.647	2.146
Total		5177501	548531	100.000	100.000

[Ala^{17}]Elevenin _LCMS

Sangon Biotech

MS Spectrum

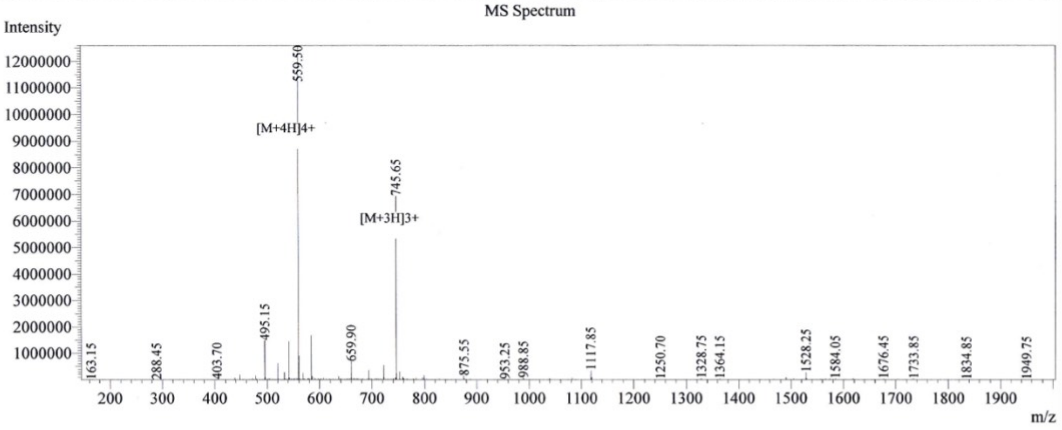

Sample Information
Dissolution method :0.1mg sample dissolved to 0.5mL by 50%ACN and 50%H2O
Date Acquired :2022/05/20 14:39:55
Injection Volume :1ul

Name :P25315-1
Sequence :RPRIDCTRFVFAPACRAVSA
Modification :C-C
Lot No. :P25315-1-220506
Theoretical :2234.644
bserved :2234.00

Interface :ESI
Nebulizing Gas Flow :1.50L/min
CDL Temp :250℃
CDL Volt :0v
Block Temp :200

Prerod Bias :+4.5kv
Detector :-0.2kv
T.Flow :0.2ml/min
B.conc :50%H2O/50%MeOH

生工生物工程（上海）股份有限公司

地址：上海市松江区香闵路698号
电话/Tel: 400-821-0268
邮箱/Email: Sales@sangon.com

Add: 698 Xiang Min Road Songjiang Shanghai China
传真/Fax: 86-21-37772170
网址/Web: wwww.sangon.com

[Ala^{18}]Elevenin_COA

Sangon Biotech 多肽合成报告单

CERTIFICATE OF ANALYSIS

**Product Name**	P27392-3
**Catalog No.**	N/A
**Lot No.**	P27392-3-23021501
**Sequence**	RPRIDCTRFVFAPACRGASA
**Length**	20AA
**Modification**	C-C
**Molecular Weight (MW)**	2192.56
**Storage**	-20°C

Test Items	Specifications	Results
**MW by MS**	2192.4	Conforms
**Purity by HPLC**	> 95%	97.804%
**Peptide Content**	N/A	N/A
**Acetic acid content**	N/A	N/A
**Appearance**	White to off-white lyophilized powde	Conforms
**Quantity**	1mg*5vials	5.0mg

Certified by: *Melinda* Date 02/28/2023

Quality Assurance Department

[Ala^{18}]Elevenin_HPLC

Sangon Biotech

Sample Information

Name	:	P27392-3
Sequence	:	RPRIDCTRFVFAPACRGASA
Modification	:	C-C
Lot.No	:	P27392-3-23021501
Pump A	:	0.1%trifluoroacetic in 100%water
Pump B	:	0.1%trifluoroacetic in 100%acetonrtrile
Total Flow	:	1.0ml/min
Wavelength	:	214nm
Analytical column type	:	SHIMADZU shim-pack GIST(4.6*250MM*5UM)
Dissolution method	:	0.1mg sample dissolved to 0.5mL by 100%H2O
Acquisition Time	:	2023/02/28 09:28:50
Inj.Volume	:	30ul

Time	Module	Action	Value
0.01	Pumps	B.Conc	21
20.00	Pumps	B.Conc	41

Chromatogram

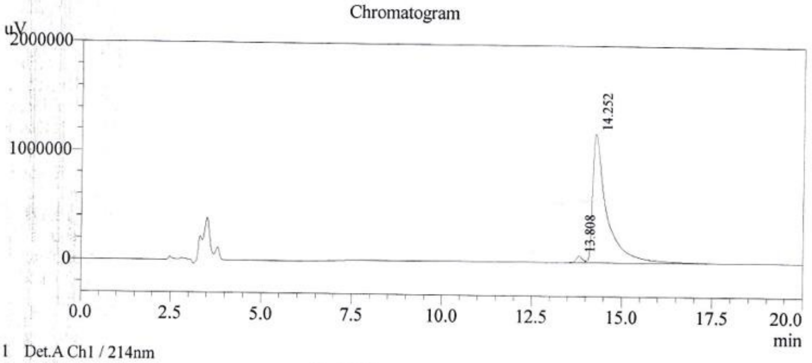

1 Det.A Ch1 / 214nm

PeakTable

Detector A Ch1 214nm

Peak#	Ret. Time	Area	Height	Area %	Height %
1	13.808	727847	59527	2.196	4.795
2	14.252	32423801	1181794	97.804	95.205
Total		33151649	1241322	100.000	100.000

生工生物工程（上海）股份有限公司

地址: 上海市松江区香闵路698号
电话/Tel: 400-821-0268
邮箱/Email: sales@sangon.com

Add: 698 Xiang Min Road SongJiang Shanghai China
传真/Fax: 86-21-37772170
网址/Web: www.sangon.com

[Ala^{18}]Elevenin_LCMS

Sangon Biotech

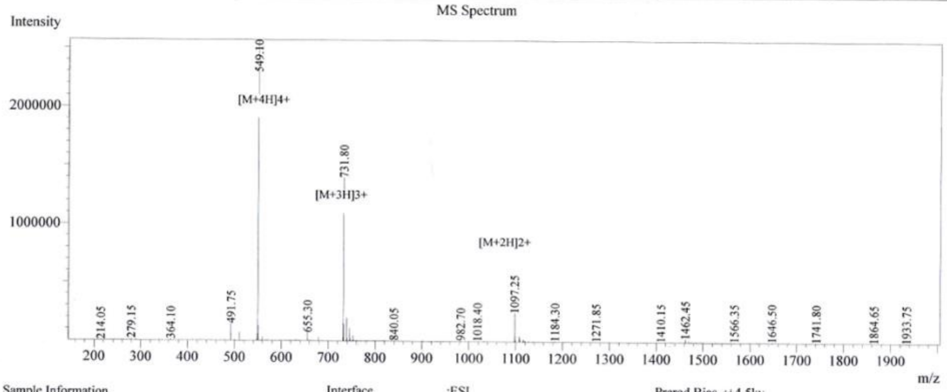

Sample Information
Dissolution method :0.1mg sample dissolved to 0.5mL by 50%ACN and 50%H2O
Date Acquired :2023/02/28 10:14:09
Injection Volume :1ul
Name :P27392-3
Sequence :RPRIDCTRFVFAPACRGASA
Modification :C-C
Lot No. :P27392-3-23021501
Theoretical :2192.563
bserved :2192.40

Interface :ESI
Nebulizing Gas Flow :1.50L/min
CDL Temp :250°C
CDL Volt :0v
Block Temp :200

Prerod Bias :+4.5kv
Detector :-0.2kv
T.Flow :0.2ml/min
B.conc :50%H2O/50%MeOH

生工生物工程（上海）股份有限公司

地址：上海市松江区香闵路698号
电话/Tel: 400-821-0268
邮箱/Email: Sales@sangon.com

Add: 698 Xiang Min Road Songjiang Shanghai China
传真/Fax: 86-21-37772170
网址/Web: wwww.sangon.com

Elevenin$_{5-17}$_COA

Sangon Biotech 多肽合成报告单

CERTIFICATE OF ANALYSIS

**Product Name**	P25315-9
**Catalog No.**	N/A
**Lot No.**	P25315-9-22051001
**Sequence**	DCTRFVFAPACRG
**Length**	13AA
**Modification**	C-C
**Molecular Weight (MW)**	1440.67
**Storage**	-20°C

**Test Items**	**Specifications**	**Results**
**MW by MS**	1440.2	Conforms
**Purity by HPLC**	> 98%	98.166%
**Peptide Content**	N/A	N/A
**Acetic acid content**	N/A	N/A
**Appearance**	White to off-white lyophilized powde	Conforms
**Quantity**	1mg*5vials	5.0mg

Certified by: Melinda   Date 05/20/2022

Quality Assurance Department

Elevenin$_{5-17}$_HPLC

Sangon Biotech

Sample Information

Name	: P25315-9
Sequence	: DCTRFVFAPACRG
Modification	: C-C
Lot.No	: P25315-9-22051001
Pump A	: 0.1%trifluoroacetic in 100%water
Pump B	: 0.1%trifluoroacetic in 100%acetonrtrile
Total Flow	: 1.0ml/min
Wavelength	: 214nm
Analytical column type	: SHIMADZU Inertsil ODS-SP(4.6*250MM*5UM)
Dissolution method	: 0.1mg sample dissolved to 0.5mL by 10%ACN and 90%H2O
Acquisition Time	: 2022/05/20 13:54:16
Inj.Volume	: 50ul

Time	Module	Action	Value
0.01	Pumps	B.Conc	20
20.00	Pumps	B.Conc	40

Chromatogram

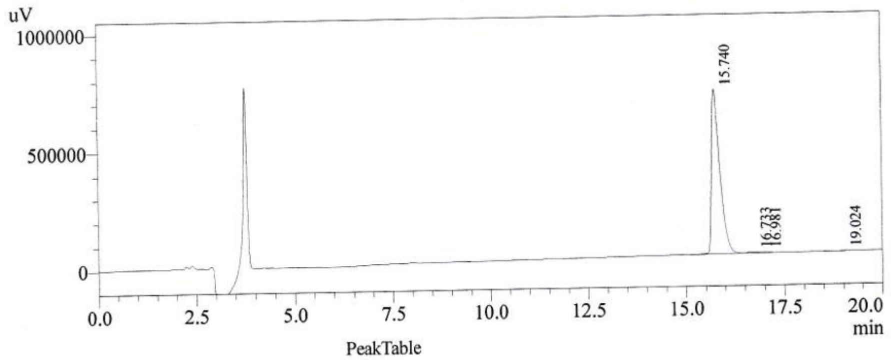

PeakTable

Detector A Ch1 214nm

Peak#	Ret. Time	Area	Height	Area %	Height %
1	15.740	10476513	696547	98.166	98.339
2	16.733	97935	5179	0.918	0.731
3	16.981	87114	5370	0.816	0.758
4	19.024	10711	1215	0.100	0.172
Total		10672274	708310	100.000	100.000

生工生物工程（上海）股份有限公司

地址: 上海市松江区香闵路698号
电话/Tel: 400-821-0268
邮箱/Email: sales@sangon.com

Add: 698 Xiang Min Road SongJiang Shanghai China
传真/Fax: 86-21-37772170
网址/Web: www.sangon.com

Elevenin$_{5\text{-}17}$_LCMS

Sangon Biotech

MS Spectrum

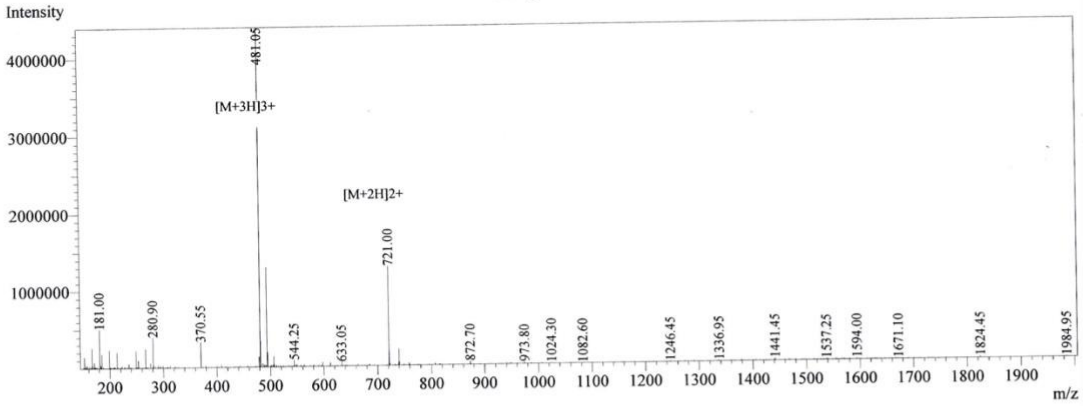

Sample Information
Dissolution method :0.1mg sample dissolved to 0.5mL by 50%ACN and 50%H2O
Date Acquired :2022/05/20 13:55:39
Injection Volume :1ul

Name :P25315-9
Sequence :DCTRFVFAPACRG
Modification :C-C
Lot No. :P25315-9-22051001
Theoretical :1440.674
bserved :1440.15

Interface :ESI
Nebulizing Gas Flow :1.50L/min
CDL Temp :250°C
CDL Volt :0v
Block Temp :200

Prerod Bias :+4.5kv
Detector :-0.2kv
T.Flow :0.2ml/min
B.conc :50%H2O/50%MeOH

生工生物工程（上海）股份有限公司

地址：上海市松江区香闵路698号
电话/Tel: 400-821-0268
邮箱/Email: Sales@sangon.com

Add: 698 Xiang Min Road Songjiang Shanghai China
传真/Fax: 86-21-37772170
网址/Web: www.sangon.com

Elevenin$_{6-15}$_COA

Sangon Biotech

多肽合成报告单

CERTIFICATE OF ANALYSIS

**Product Name**	P25315-5
**Catalog No.**	N/A
**Lot No.**	P25315-5-220506
**Sequence**	CTRFVFAPAC
**Length**	10AA
**Modification**	C-C
**Molecular Weight (MW)**	1112.35
**Storage**	-20°C

**Test Items**	**Specifications**	**Results**
**MW by MS**	1111.8	Conforms
**Purity by HPLC**	> 98%	98.299%
**Peptide Content**	N/A	N/A
**Acetic acid content**	N/A	N/A
**Appearance**	White to off-white lyophilized powde	Conforms
**Quantity**	1mg*5vials	5.0mg

Certified by: Melinda   Date 05/16/2022

Quality Assurance Department

Elevenin$_{6\text{-}15}$_HPLC

Sangon Biotech

Sample Information

Name	:	P25315-5
Sequence	:	CTRFVFAPAC
Modification	:	C-C
Lot.No	:	P25315-5-220506
Pump A	:	0.1%trifluoroacetic in 100%water
Pump B	:	0.1%trifluoroacetic in 100%acetonrtrile
Total Flow	:	1.0ml/min
Wavelength	:	214nm
Analytical column type	:	SHIMADZU Inertsil ODS-SP(4.6*250MM*5UM)
Dissolution method	:	0.1mg sample dissolved to 0.5mL by 20%ACN and 80%H2O
Acquisition Time	:	2022/05/16 11:53:42
Inj.Volume	:	50ul

Time	Module	Action	Value
0.01	Pumps	B.Conc	29
20.00	Pumps	B.Conc	49

Chromatogram

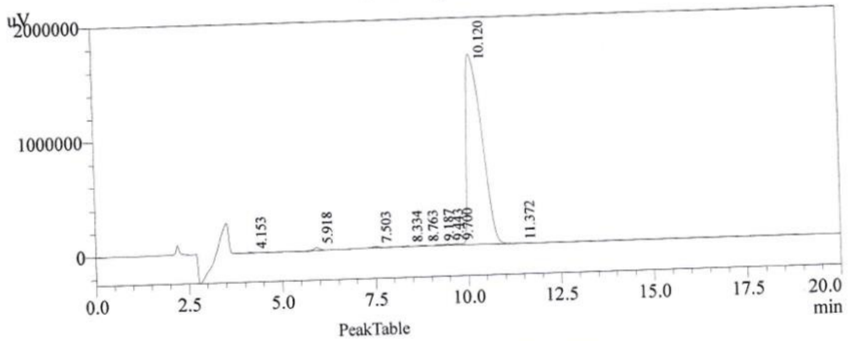

PeakTable

Detector A Ch1 214nm

Peak#	Ret. Time	Area	Height	Area %	Height %
1	4.153	36588	4912	0.076	0.284
2	5.918	271866	24467	0.562	1.414
3	7.503	140145	8563	0.289	0.495
4	8.334	32229	3638	0.067	0.210
5	8.763	44342	3306	0.092	0.191
6	9.187	65413	6724	0.135	0.388
7	9.443	89661	7731	0.185	0.447
8	9.700	140627	11112	0.290	0.642
9	10.120	47587068	1660134	98.299	95.914
10	11.372	2677	268	0.006	0.015
Total		48410615	1730855	100.000	100.000

生工生物工程（上海）股份有限公司

地址: 上海市松江区香闵路698号
电话/Tel: 400-821-0268
邮箱/Email: sales@sangon.com

Add: 698 Xiang Min Road SongJiang Shanghai China
传真/Fax: 86-21-37772170
网址/Web: www.sangon.com

Elevenin$_{6\text{-}15}$_LCMS

Sangon Biotech

MS Spectrum

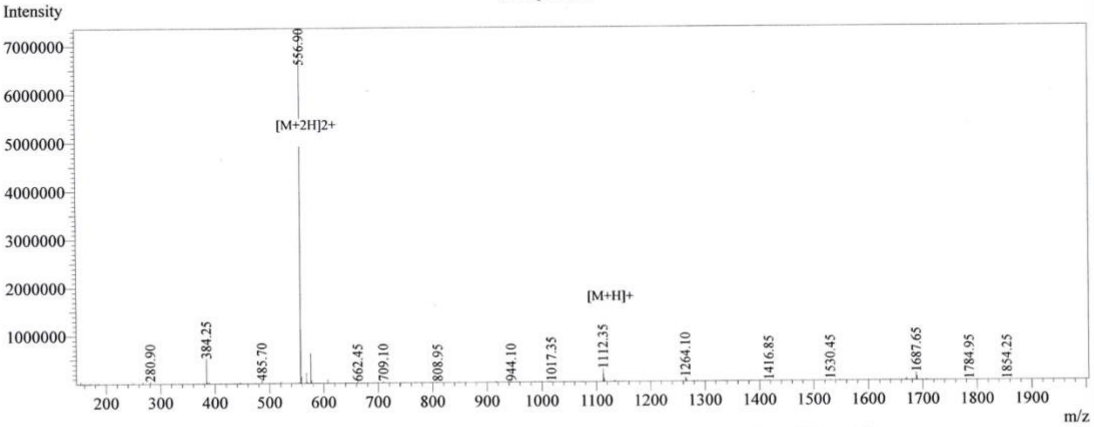

Sample Information
Dissolution method :0.1mg sample dissolved to 0.5mL by 50%ACN and 50%H2O
Date Acquired :2022/05/16 12:10:02
Injection Volume :1ul

Interface :ESI
Nebulizing Gas Flow :1.50L/min
CDL Temp :250°C
CDL Volt :0v
Block Temp :200

Prerod Bias :+4.5kv
Detector :-0.2kv
T.Flow :0.2ml/min
B.conc :50%H2O/50%MeOH

Name :P25315-5
Sequence :CTRFVFAPAC
Modification :C-C
Lot No. :P25315-5-220506
Theoretical :1112.345
bserved :1111.80

生工生物工程（上海）股份有限公司

地址：上海市松江区香闵路698号
电话/Tel: 400-821-0268
邮箱/Email: Sales@sangon.com

Add: 698 Xiang Min Road Songjiang Shanghai China
传真/Fax: 86-21-37772170
网址/Web: wwww.sangon.com
